# Reported problems and responses during the conduct of stepped-wedge cluster randomized trials in healthcare settings: a qualitative systematic review

**DOI:** 10.1093/ije/dyaf217

**Published:** 2026-01-02

**Authors:** Kathryn S Taylor, Julie McLellan, Caroline Kristunas, Clare Bankhead, Nicola Pidduck, Nia W Roberts, Rafael Perera, Karla Hemming

**Affiliations:** Nuffield Department of Primary Care Health Sciences, University of Oxford, Oxford, United Kingdom; Nuffield Department of Primary Care Health Sciences, University of Oxford, Oxford, United Kingdom; Institute of Cancer and Genomic Sciences, University of Birmingham, Birmingham, United Kingdom; Nuffield Department of Primary Care Health Sciences, University of Oxford, Oxford, United Kingdom; Nuffield Department of Primary Care Health Sciences, University of Oxford, Oxford, United Kingdom; Bodleian Health Care Libraries, University of Oxford, Oxford, United Kingdom; Nuffield Department of Primary Care Health Sciences, University of Oxford, Oxford, United Kingdom; Department of Public Health, Epidemiology and Biostatistics, University of Birmingham, Birmingham, United Kingdom

**Keywords:** stepped-wedge cluster randomized trial, qualitative review, real-world problems, recruitment, implementation, protocol adherence, methodology

## Abstract

**Background:**

The stepped-wedge cluster randomized trial (SW-CRT) is a pragmatic complex design that can be difficult to implement. We aimed to summarize the reported problems and responses to problems in studies recently published after the publication of the reporting guidelines for SW-CRTs.

**Methods:**

We searched the literature for SW-CRTs published between 9 November 2018 and 23 February 2021 to identify reported SW-CRT-related problems (defined as relating to the components of the design, i.e. involving clusters and the staggered intervention implementation) and responses to problems. We carried out a thematic analysis to derive descriptive themes and overarching analytical themes.

**Results:**

Among 84 included SW-CRTs, 62 (74%) reported 107 problems related to the SW-CRT design and 38 responses to 36 problems were reported by 24 trials. The “problems” formed six descriptive problem themes: “participant recruitment,” “cluster issues” (e.g. cluster merger or dropout), “internal factors” (e.g. logistic or administrative issues), “external factors” (e.g. weather or religious events), “outcome measurement” (e.g. practicalities around measurement of repeated outcomes), and “intervention implementation” (e.g. delays or contamination). The “responses” formed six descriptive themes: “adding new clusters,” “modifying the randomization,” “reducing contamination,” “changing outcomes,” “intention-to-treat,” and “modifying the analysis.”

**Conclusion:**

SW-CRTs commonly run into problems. Two overarching and conflicting analytical problem themes emerged: the “struggle to adhere to the protocol,” given the defining features of the SW-CRT design, when faced against “real-life pressures” created by internal and external factors. Further research is needed to explore whether responses to these problems have resource or integrity ramifications.

Key Messages

- Stepped-wedge cluster randomized trial (SW-CRT) studies are difficult to implement, but the types of problems encountered and how these problems may be addressed have not been systematically reviewed.
- Researchers are conflicted between trying to adhere to the protocol and dealing with real-life pressures.
- Further research is necessary to evaluate whether reported responses to problems can form formal guidance for those interested in conducting SW-CRT studies.

## Introduction

The stepped-wedge cluster randomized trial (SW-CRT) has become increasingly popular in health and social care in recent years [1]. There is rising pressure on commissioners and payers of health and social services to invest in complex interventions and adopt them with a rigorous evidence base [2]. The evaluation of complex interventions, particularly those in implementation research, is often pragmatic and the desire to consider effects in the context of “real-world” rollouts [3]. Moreover, due to the nature of complex interventions, randomization at the level of the individual is often inappropriate [2]. The SW-CRT study design is often appealing in these settings. In a SW-CRT, all clusters start as controls and the intervention is then introduced gradually at regular intervals (“the steps”) to a single cluster or group of clusters by using randomization.

The SW-CRT is a relatively novel study design and many aspects of it are still in development. This is reflected in the multitude of systematic reviews about different aspects of this study design, including its conduct and reporting, and discussions about its potential benefits as well as limitations [1, 4–16]. Indeed, whilst the SW-CRT has many perceived advantages, there are logistical challenges. Researchers have highlighted problems and challenges with this study design, including maintaining the implementation schedule, contamination between and within clusters, under-recruitment, confounding by secular changes in the outcome, and missing data [14, 17–22]. However, these studies have various limitations. For example, some are general discussions [17, 18] or experiences of single trials [20, 21] or a review of a few trials [19]. While some systematic reviews [14, 22] have explored recruitment and implementation problems, focusing on the prevalence of problems, none has qualitatively evaluated the underlying problem themes or explored how trialists have responded to problems encountered during SW-CRTs. Furthermore, while the recent publication of the Consolidated Standards of Reporting Trials (CONSORT) extension to SW-CRTs in November 2018 provides reporting guidelines [23], there is no single point of reference offering practical advice for researchers conducting SW-CRTs on how to address problems that arise [22].

Therefore, as a first step towards developing useful practical advice, we aimed to undertake a qualitative systematic review of SW-CRTs published after November 2018, to identify and describe reported SW-CRT problems encountered whilst undertaking this design and the responses to these problems.

## Methods

The study protocol was registered with the Open Science Framework (10.2.21, doi 10.17605/OSF.IO/FVSG4). This systematic review is reported in line with recommendations by the Preferred Reporting Items for Systematic reviews and Meta-Analyses [24] and Enhancing Transparency in Reporting the Synthesis of Qualitative Research [25] guidelines. The completed checklists are reported in Supplementary Tables S1–S3.

### Search strategy and eligibility criteria

Our search strategy and eligibility criteria are described in the Supplementary Methods.

### Study selection

Pairs of reviewers (K.T., J.M., N.P., C.K., and K.H.) screened the search results independently. Disagreements were resolved by discussion or referral to another reviewer.

### Data extraction and management

A new data-extraction form was initially trialed for five studies. Data extraction was performed independently by pairs of reviewers (K.T., J.M., N.P., C.K., and K.H.), data were crosschecked, and disagreements were resolved by discussion or referral to another reviewer. For all included SW-CRT studies, we located any referenced and published study documents (e.g. trial protocol) and checked them for information about the trial and problems that arose.

#### Descriptive characteristics

To describe the studies, extracted data included: factors relating to the study’s context, namely the study setting, study area, country, and publication year; the rationale for using the stepped-wedge design; and whether or not the trial was a pilot or feasibility study or whether such a study had been carried out previously. We also extracted the number of participants analysed (analysis sample size), the target sample size, and several key study design features as outlined by Copas *et al.* [26] comprising the number of clusters, number of sequences, design types for data collection (complete or incomplete, e.g. with a transition period) and recruitment (continuous, closed cohort, or open cohort; see Supplementary Fig. S5 for more details), and method of measurement of the primary outcome (repeated measurements from individuals, cross-sectional at fixed calendar times, single measurement at a certain time after exposure or time from exposure to an event, or number of events in an exposure period).

#### Reported problems and responses to problems

We defined SW-CRT-related problems as: (i) relating to the key components of the SW-CRT design (i.e. staged implementation) or affecting clusters and (ii) encountered during the study. Some problems were included that were not reported explicitly, such as intervention implementation delays, where these were apparent from the reported SW-CRT design diagram; and recruitment problems, e.g. where it was clear that the recruitment target was not met. We excluded SW-CRT-related problems that the authors speculated on in a discussion but for which there was no clear report of any problem (e.g. speculation that a perceived lack of fairness about waiting for the intervention might have impacted recruitment or retention, but there were no reported material consequences such as lower recruitment [27]). We also excluded non-SW-CRT-related problems that occurred during a SW-CRT (e.g. missing outcome data that were not clearly related to the SW-CRT aspect [28–30]). We defined a response to a SW-CRT-related problem as any reported effort to address the reported SW-CRT problem, or explicitly ignore it, during the study.

Data were extracted on the broad categorization of the reported problems and/or response, such as recruitment, intervention implementation, or contamination between clusters, as well as a verbatim quotation of the description of the problem and/or response. To identify participant recruitment problems, we also compared the sample-size target with the number of participants included in the analysis of the primary outcome and, if the analysis number was >10% under the target, we inferred an under-recruitment problem.

### Data analysis

Descriptive analysis was carried out to summarize the characteristics of the studies. We adapted the thematic synthesis methods of Thomas and Harden [31], applying these methods only to text quotations of reported problems and responses, as opposed to the analysis of the whole article. The extracted text underwent semantic coding sentence by sentence inductively. The descriptive codes were then grouped, by using an iterative process (starting with the grouping as initial categorizations, as described above), into descriptive themes to summarize the qualitative data. These themes were interpreted to derive overarching analytical themes. This iterative process was conducted through discussion between four reviewers (K.T., J.M., K.H., and C.K.). This process was applied independently to both the “problems” and the “responses.”

## Results

### Included studies

**Figure 1.**
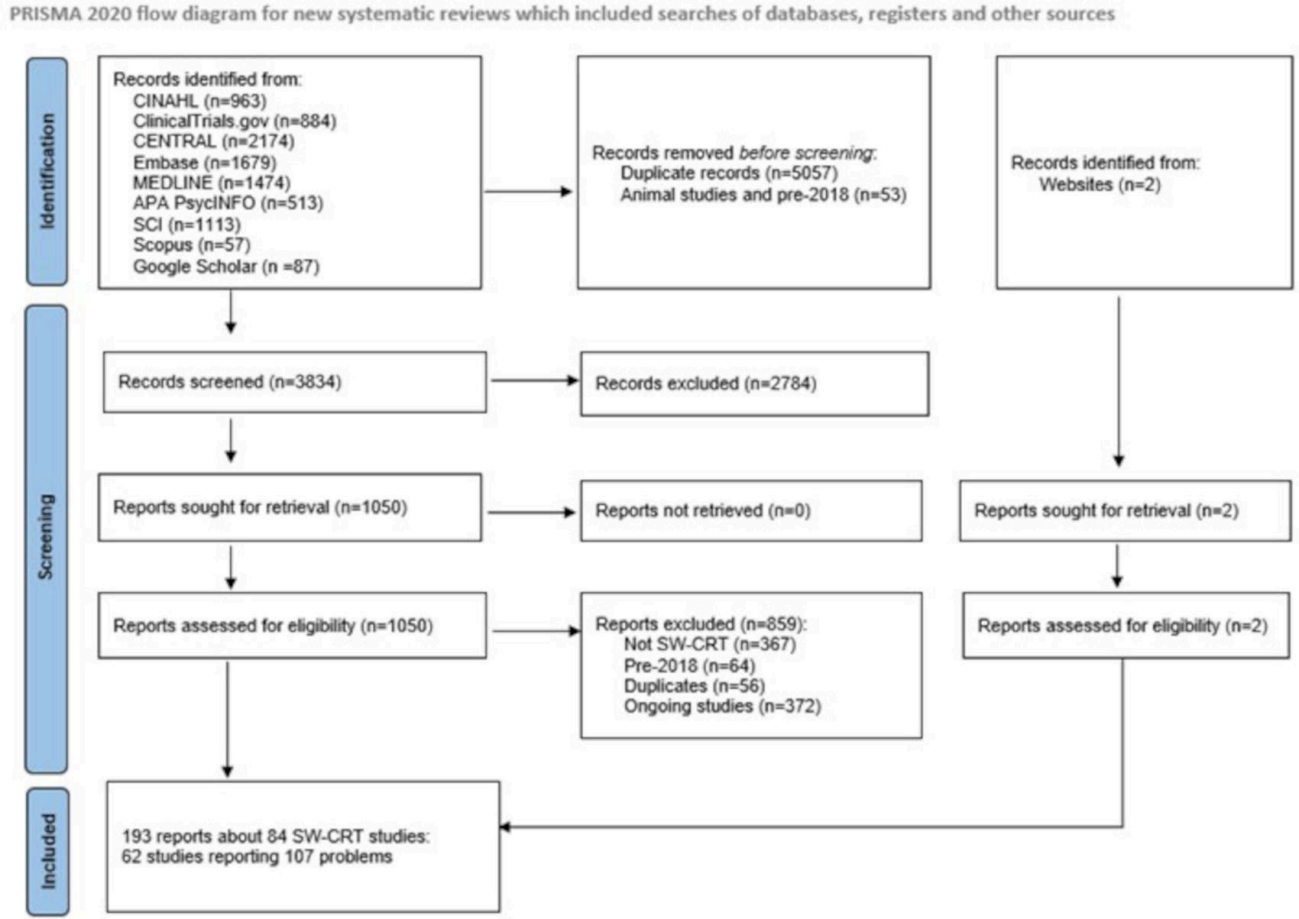
Flow chart showing study selection. CENTRAL, Cochrane Central Register of Controlled Trials; CINAHL, Cumulated Index to Nursing and Allied Health Literature; SCI, Science Citation Index. The World Health Organization International Clinical Trials Registry Platform (WHO ICTRP) was not accessible at time of search.

From 3836 records identified from databases and websites, we included 84 SW-CRTs (Fig. 1). They were described by 193 reports that were a single full study report, a protocol and a full study report, or a protocol and reports of primary and secondary results. All papers were published in English. For each study, the report providing the most information is cited in the references listed in Supplementary Table S4.

Table 1 summarizes the study characteristics of the 84 studies, with more details provided in Supplementary Table S5. Forty-seven studies (56%) were conducted in hospitals, with infectious diseases or hand hygiene the most common study area (24 studies, 29%). Forty-five studies (54%) were based in Europe or North America. Seven (8.5%) were pilot or feasibility studies and 26 (31%) reported having a prior feasibility or pilot study to test at least some aspects of the SW-CRT design. Seventy studies (83%) used a complete design type for data collection and 14 (17%) used an incomplete design, e.g. with a transition period. Fifty-seven (70%) had continuous recruitment. Outcomes were most often measured by using single measurements (35 studies, 42%) and 18 (21%) studies had a repeated-measurements design.

**Table 1.** Overview of characteristics of the included studies.

Characteristic	*N* (%) or median (interquartile range) (*N* = 84)
Setting	
Hospital	47 (56)
Primary or community care	27 (32)
Residential care	6 (7)
Other	4 (4)
Area of study	
Infectious diseases/hand hygiene	24 (29)
Obstetrics	13 (16)
Cardiovascular	9 (11)
Healthcare administration/general health issues	7 (8)
Other medical specialties	31 (37)
Location of study recruitment	
Europe	23 (27)
North America	22 (26)
Oceania	10 (12)
Africa	14 (17)
Asia	11 (13)
South America	3 (4)
Africa/Asia/North America	1 (1)
Publication year	
2018	5 (6)
2019	34 (41)
2020	41 (49)
2021	4 (5)
Rationale for stepped-wedge design (*n* = 69, not mutually exclusive)	
All sites receive intervention	43 (51)
Need for staggered implementation	41 (49)
Statistical reasons	41 (49)
Ethical or equity reasons	13 (16)
Acceptability	10 (12)
Other reason	11 (13)
Use of pilot or feasibility study	
Current study was reported as a pilot/feasibility	7 (9)
Prior pilot/feasibility was reported	26 (33)
Trial features	
Number of clusters	10 (6, 18)
Number of sequences	4 (3, 5)
Data-collection design	
Complete	70 (83)
Incomplete	14 (17)
Recruitment design (*n* = 80)	
Continuous recruitment	57 (70)
Closed cohort	13 (16)
Open cohort	10 (12)
Primary outcome measurement design (*n* = 83)	
Repeated, at fixed times	8 (10)
Repeated, linked to the exposure time	10 (12)
Cross-sectional, at fixed calendar time(s)	16 (19)
Single measurement, linked to exposure time	35 (42)
Time to event, where time begins at the start of exposure	1 (1)
Number of events in an exposure period	6 (16)

### Reported problems

Among the 84 included studies, 62 (74%) reported 107 problems related to the SW-CRT design (Supplementary Table S6). Of these 62 studies, five were pilot or feasibility studies and 22 reported having carried out a pilot or feasibility study beforehand, including eight designed specifically to test the SW-CRT design (Supplementary Table S5). The SW-CRT-related problems formed six descriptive problem themes (Table 2).

**Table 2.** Descriptive themes and codes for reported SW-CRT-related problems.

Descriptive theme	Explanation	Codes	Studies
Participant recruitment	Unexpected recruitment levels, or variation in recruitment or eligibility either between clusters or between control and intervention phases or overall, or problems obtaining consent or engagement with the participant recruiters	**Sub-theme: Imbalance across clusters or treatment conditions**	
		Imbalance in eligible cases across the clusters between control and intervention	DECIDE LVAD
		Imbalance in participant recruitment between clusters	EvANtiPain, PAINRelieveIT Hospice, POC-EID, SAMBA
		Reduced participant engagement due to frustration of having to wait for the intervention	McGuiness 2020
		Lower participant recruitment in one or more clusters	ELIZABETH, CATH TAG, CRADLE-3, EPOCH, Fasugba 2019, ICAN QUIT in pregnancy, Mazurek 2020, Naser 2020, PACT-HF, Pro-Motion, REMAIN HIOME, Schnipper 2021, Silverberg 2020, SO-HIP, Trent 2019, Worster 2020, XPRES Williams
		Lower participant recruitment in the control phase	IRIS, SAMBA
		Lower participant recruitment, particularly in the intervention phase	EvANtiPain
		None or lower participant recruitment in the intervention phase for one or more clusters	CSNAT-1, PAINRelieveIT Hospice, POC-EID
		Higher participant recruitment in later steps	PARROT
		Higher participant recruitment in one or more clusters	PAINRelieveIT Hospice, Graham 2019
		**Sub-theme: Lower participant recruitment**	
		Low participant numbers	Graham 2019
		Lack of engagement with participant recruiters	Healthy Hearts NYC
		Lower participant recruitment due to competition from a new external facility	PAINRelieveIT Hospice
Cluster issues	Unanticipated loss or merger of clusters, or variation in cluster size or prevalence of outcomes	One or more clusters closed	PHRASYL, Graham 2019
		One or more clusters dropped out	FallDem, IRIS, LIRE, CPACS-3, STRIVE, Aguis 2020, Healthy Hearts NYC
		Two or more clusters merged	EPOCH, PAINRelieveIT Hospice
		Cluster definition unclear due to poor linkage between observations and cluster	Worster 2020
		Wanting to add more clusters after randomization	PERCEIVE
		Cluster-size variation	CSNAT-I, Graham 2019
		Higher-than-expected intra-cluster correlation	Graham 2019
		Lower-than-expected intra-cluster correlation	PARROT
Internal factors	Logistical, operational, administrative, and personnel factors, affecting one or more clusters, that the trialists were unable to anticipate, control or avoid, which impacted the study. This includes the loss, change, and limitation of staff and resources, and the engagement of staff in the study	Logistical/operational/administrative problems delayed the intervention rollout in one or more clusters	Making it Happen, Mazurek 2020, NePeriQIP, Pradam 2019, SPEC, THISTLE, Worster 2020
		Logistical/operational/administrative problems brought forward the intervention rollout in one or more clusters	SPEC
		Logistical problems delayed the study start	Worster 2020, STRAP
		Approval delays led to a delayed intervention rollout in one or more clusters	Mazurek 2020
		Electronic health record system change in one or more clusters temporarily halted recruitment	DOSE-NPV
		Lack of funding and resources led to inconsistent implementation across clusters	THISTLE
		Staff shortages, staff constraints, or skill-mix changes led to inconsistent implementation in one or more clusters	Barrera 2019, Haines 2020
		Staff shortages, staff constraints, or skill-mix changes caused recruitment challenges in one or more clusters	Healthy hearts NYC
		Staff shortages, staff constraints, or skill-mix changes delayed the intervention rollout in one or more clusters	Pradam 2019, Schnipper 2021
		Staff constraints disrupted the intervention rollout	CRADLE-3
		Staff shortages/skill-mix changes in one or more clusters contributed to one or more clusters dropping out	FallDem
		Staff working in multiple clusters due to resource constraints	DART, Training for life
		Medical-services limitation of one or more clusters	CPACS-3
		Waning interest due to the data-collection burden	Silverberg 2020
		Frustration of having to wait for the intervention affected staff turnover	Healthy Hearts NYC
		Shortened study duration due to the electonic health record system change in one or more clusters that temporarily halted data collection	Trent 2019
		Study duration was extended due to under-recruitment	CSNAT-I
		Study duration was extended due to understaffing over the Christmas period	Keogh 2020
		Study duration was extended due to computing systems failure and temporary intervention loss in some clusters	Selby 2019
		Study duration was extended due to the paused implementation in some clusters when lower recruitment expected	EvANtiPain
External factors	Factors and events affecting one or more clusters that the trialists were unable to anticipate, control, or avoid, that impacted the study. This includes issues of security, religion, seasonality, weather, disease prevalence, competition, and national clinical practice improvements	Religious event in the control period shortened the study duration	CHIME
		Intervention device update led to inconsistent intervention implementation	XPRES
		Severe weather event delayed the study start	McGuiness 2020
		Severe weather event affected the prevalence of outcomes in some clusters	SMARThealth India
		Environmental problems in one or more clusters disrupted the intervention rollout	Naser 2020
		Environmental problems in one or more clusters led to delays that extended the study duration	McGuiness 2020
		Seasonal variation in the prevalence of the disease/condition led to the study being extended	ELIZABETH, STRAP
		Security concerns in one or more clusters led to the loss of clusters	Aguis 2020
		Industrial action in one or more clusters disrupted the intervention rollout	NePeriQIP, Graham 2019
		Industrial action in one or more clusters led to staff constraints	CRADLE-3
		Production and delivery delays extended the study duration	SmartRub
		National guideline changes/differences in one or more clusters affected implementation	MaxART, DOSEM HPV, Mazurek 2020, ORCAS
		Competition from a new external facility reduced recruitment in one or more clusters	PAINReliefIT Hospice
		A natural disaster disrupted data collection	CRADLE-3
Outcome measurement	Concerns about the practicalities of outcome measurement relating to the crossover points or variation between clusters, or problems with the frequency of data collection in stepped-wedge-design trials	Variability across clusters of timing of the post-intervention survey	ICAN QUIT in pregnancy
		Accuracy of timing of data collection at crossover points	CATH TAG
		Measurement burden related to purposive data-collection methods	Haines 2020
		Measurement bias due to frequent data collection by care staff	FallDem
		Some participants left the cluster before outcome data were measurable	CPACS-3
		One or more clusters did not provide intervention data as time ran out	Making it Happen
Intervention implementation	Intervention implementation problems involving the receipt, timing of the delivery of the intervention over time or contamination due to staff discussions between clusters	**Sub-theme: Variation of the dose of the intervention across clusters**	
		Increased awareness of intervention over time in one or more clusters	PARROT
		Inconsistent intervention implementation across clusters	Barrera 2019, Haines 2020, Mazurek 2020, PovuPoa School, Pradam 2019, Rikin 2020, SOCLE II, THISTLE, XPRES, Selby 2019, MaxART
		Some clusters had a duration for the implementation of the intervention	Leis 2020
		Absenteeism and lack of consent prevented implementation	Barerra 2019
		Refusal to implement the intervention by one or more clusters	THISTLE
		Partially implemented intervention in one or more clusters	Haines 2020
		Actual practice change was longer than the planned transition period	Graham 2019
		Low intervention uptake in some clusters due to the frustration of having to wait for the intervention	McGuiness 2020
		One or more clusters did not implement the intervention	Naser 2020, THISTLE
		Frustration of having to wait for the intervention reduced intervention uptake	McGuiness 2020
		**Sub-theme: Deviations from the randomization schedule**	
		Intervention training times not consistent with intervention implementation	Shah 2020
		Intervention training in control period	Shah 2020
		One cluster was exposed to the intervention before the study	THISTLE
		One or more clusters were exposed to the intervention for all steps	Making it Happen, Shekhawat 2020
		Some participants were admitted during the (wash-in) transition period and were switched to intervention earlier than the rest of the cluster	Graham 2019
		Intervention rollout delayed or brought forward in one or more clusters	SPEC, CSNAT-I, IRIS, ORCAS, Pradham 2019, Worster 2020, DIZZINCT, SAMBA, Williams 2019, Making it Happen, CHIME, Schnipper 2021, NePeriQIP, THISTLE, SmartRub, Keogh 2020, Haines 2020, DOSE HPV
		**Sub-theme: Contamination and diffusion of treatment effect**	
		Staff discussion between clusters leading to contamination	Trent 2019, DART, DIZZINCT, Wong 2020
		Diffusion of treatment effect due to dissemination of intervention	NePeriQIP

The first theme is “participant recruitment,” which refers to unexpected recruitment levels, variation in recruitment or eligibility, or problems with obtaining consent or engagement with the participant recruiters. Two sub-themes emerged within the “participant recruitment” problem theme. The first is “imbalance across clusters or treatment conditions,” such as the recruitment imbalance between clusters that occurred during the SAMBA [32] and IRIS [33] trials, which had lower participant recruitment in the control phase. Another example is a trial in which the authors reported that the last cluster to receive the intervention experienced frustration with waiting and became less engaged with the participant recruiters [34]. The second sub-theme is “lower recruitment,” which is more generally not clearly differential across either clusters or time. Examples include the XPRES [35] trial, in which the number of participants meeting the eligibility criteria was lower than expected across all sequences and periods, and this resulted in a much lower total sample size than anticipated at the end of the study.

The second theme is “cluster issues,” which is about the cluster-specific features of the SW-CRT design, such as the unanticipated loss or merger of a cluster, or variation in cluster size or prevalence of outcomes. Examples include the trial in which four clusters left the study due to security concerns [36]; the EPOCH trial, in which some clusters merged during the study [27]; and the PARROT trial, in which the intra-cluster correlation was lower than expected [37].

The third and fourth themes, “internal factors” and “external factors,” cover a host of logistical, operational, or administrative problems that the trialists were unable to anticipate, control, or avoid. This led to a shorter study duration in two studies [38, 39]. “Internal factors” are internal to the trial (not caused by external events beyond the control of the investigators). Examples include competing practice priorities producing staff shortages in the Healthy Hearts NYC trial [40] and approval delays in the trial reported by Mazurek *et al.* [41]. “External factors” refers to external causes, such as the implementation delays due to the study period of the CHIME trial coinciding with Ramadan [38]. Another example is the disruption to the trial that arose due to heavy monsoonal flooding [34].

The fifth theme, “outcome measurement,” relates to the practicalities and frequency of outcome measurement in SW-CRT trials. Examples include the problems that arose in the CATH TAG trial [42], in which some of the measurements were made in the predefined windows of measurement, and in the Making it Happen trial [30], which ended before the outcome data could be collected for the final sequence.

The sixth descriptive problem theme is “intervention implementation,” which refers to problems involving the receipt and timing of the delivery of the intervention over time or contamination due to staff discussions between clusters. This theme has three sub-themes. The first is “variation of the dose of the intervention across clusters,” such as in the THISTLE trial, in which two clusters refused to implement the intervention and thus remained in the control phase for all periods in the trial [21], and in another trial, in which the implementation of the intervention was longer than the planned transition period [43]. Second are “deviations from the randomization schedule,” such as in the CSNAT-I trial, in which some clusters crossed over early to the intervention to boost recruitment [44], and in the DOSEHPV trial, in which the installation of a new electronic medical record system led to delays in supporting some clusters crossing over to the intervention phase [45]. The final sub-theme is “contamination and diffusion of treatment effect,” such as in the DIZZINCT trial, in which staff worked at multiple clusters [46].

**Figure 2.**
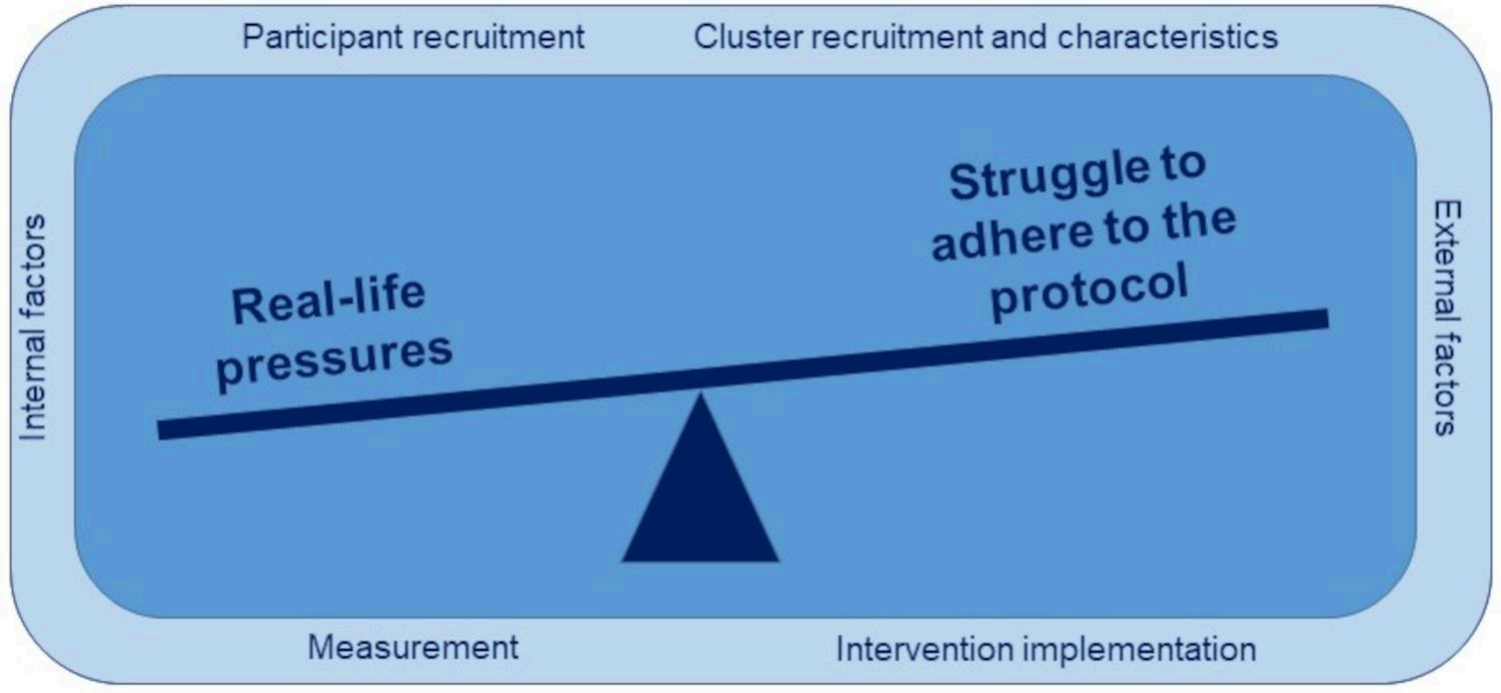
Schematic representing the descriptive problem themes and two overarching analytical themes.

Two analytical problem themes overarch the six descriptive themes: “real-life pressures” and the “struggle to adhere to the protocol.” These two themes conflict with each other (Fig. 2). They arise as problems relating to cluster and participant recruitment, the staged implementation of the intervention, and the practicalities of outcome measurement at crossover points and over time—the defining features of the SW-CRT design—which present challenges with adhering to the SW-CRT protocol. This is intensified when faced with real-life pressures from dealing with internal and external factors.

### Reported responses to SW-CRT problems

Thirty-eight responses to SW-CRT-related problems were reported by 24 studies. Each response is listed with the corresponding problem in Supplementary Table S7. These responses form six descriptive themes: “adding new clusters,” “modifying the randomization,” “reducing contamination,” “changing outcomes,” “intention-to-treat,” and, the most common theme, “modifying the analysis” (Table 3).

**Table 3.** Descriptive themes for responses to SW-CRT problems.

Descriptive theme	Explanation	Responses	Studies
Adding new clusters	Adding new clusters to compensate for the loss of clusters or to increase the total number	Added new clusters to the end of the study in a further randomization wave	PERCEIVE, ORCAS
		Added substitute clusters that were selected randomly	Aguis 2020
Modifying the randomization	Efforts in which the trialist intervened, making changes to the randomization schedule	Modified randomization to allow some clusters to have earlier crossover points and finish the study earlier	Leis 2020
		Modified randomization to allow the cluster with a lower event rate to spend more time in the intervention	DECIDE LVAD
		Intervention rollout was delayed in some clusters to boost recruitment in the control phase	IRIS
		Some clusters crossed over early to the intervention to boost recruitment	CSNAT-I
		Paused implementation when expected lower recruitment and then extended the study to compensate	EvANtiPain
		Modified the definition of the cluster in response to contamination	DART
		Cluster was allocated to a sequence in a nonrandomized way	THISTLE
Reducing contamination	Efforts made during the trial to reduce contamination	Reduced the duration of the rollout period	Shah 2020
		Instructed staff not to discuss the intervention with those working at other clusters	Trent 2019
		Included a transition period (did not include outcomes in that period)	Graham 2019
Changing outcomes	Changes to the pre-specified outcomes	Changed the primary outcome	CATH TAG
Modifying the analysis	Post hoc modifications to the analysis including changes to the analysis model or data, or conducting per-protocol, available-data, or sensitivity analyses	**Sub-theme: Changing the analysis model**	
		Did not adjust for time trends	EvANtiPain
		Used an instrumental variable analysis	Haines 2020
		Added a cluster random effect in the primary analysis	DECIDE LVAD
		**Sub-theme: Excluding or modifying data**	
		Excluded data from the period when the intervention was not available	Selby 2019
		Excluded participants admitted during a 2-week “wash-in” transition period	Graham 2019
		For clusters joining the trial late, control data were obtained from an appropriate cross-sectional study	PERCEIVE
		Excluded the cluster that had no control period in the main analysis	Shekhawat 2020
		**Sub-theme: Available case analysis**	
		Included data available	Making it Happen
		Included clusters in the analysis up to the point at which they merged	EPOCH
		Included data from the extended pre-rollout period in the main analysis	STRAP
		**Sub-theme: Sensitivity analysis**	
		Truncated the extended pre-rollout period in a sensitivity analysis	STRAP
		Excluded the cluster that had used a different screening guideline in a sensitivity analysis	Mazurek 2020
		Removed four periods of data in a sensitivity analysis when external factors disrupted the intervention implementation	CRADLE-3
		Excluded the cluster random effect in a sensitivity analysis	DECIDE LVAD
		Clusters that did not receive the intervention were considered in the control phase as a sensitivity analysis	Naser 2020
		Accounted for the implementation delays in a sensitivity analysis	NePeriQIP
		Conducted an “as implemented” sensitivity analysis	THISTLE
		Included the cluster that had no control period in a sensitivity analysis	Shekhawat 2020
Intention-to-treat	Deviations from the randomization were ignored in the analysis	Data for the clusters that did not receive the intervention were analysed according to the randomization schedule	THISTLE
		Data for the clusters that did not receive the intervention were analysed as if they had	Naser 2020
		Ignored some clusters having an earlier crossover point	Leis 2020
		Ignored the delay in the crossover points for two clusters in the main analysis	Mazurek 2020
		Ignored the differences in screening guidelines between clusters	Mazurek 2020
		Ignored the implementation delays	NePeriQIP

## Discussion

### Main findings

Over 100 SW-CRT problems were reported by studies included in our systematic review. Problems affected recruitment, cluster numbers, outcome measurement, and intervention implementation, and these problems involved various external and internal factors, which affected staffing, logistical, operational, and administrative aspects of the trials. Trialists were often unable to anticipate, control, or avoid problems, as demonstrated by several SW-CRT trialists who experienced problems despite having conducted a pilot or feasibility study beforehand to test the SW-CRT design. Two key interrelated analytical problem themes emerged: “real-life pressures” and “struggle to adhere to the protocol.” About a third of authors who reported SW-CRT problems explained how they dealt with them and most of these responses involved making post hoc changes to the analysis.

### Research in context

The complexity of conducting SW-CRT studies in terms of recruitment and implementation challenges has been previously reported in another post-CONSORT 2022 review [22]. That review focused on the prevalence of problems, whereas our review aimed to explore the types of problems and the responses through qualitative analysis. Several studies did not report information about their analysis sample size or sample-size target. Of those that reported problems, 40% did not explain how they responded. The prevalence of poor reporting of SW-CRTs has been highlighted before and after the CONSORT reporting guidelines [5, 22]. Our review has identified not only that implementation challenges are common and varied in nature, but also that full and clear reporting of challenges can aid future researchers undertaking similar designs.

The scarcity of feasibility or pilot studies for SW-CRTs has also been reported previously [13] and our review concurs with this finding, particularly the very low number of studies that conducted prior studies to test the SW-CRT design. Moreover, when analysing data from SW-CRTs, assumptions have to be made concerning secular trends [47], but these are not testable and sometimes even not expected to be tenable, as in the DOSE-NPV study [45].

### Strengths and limitations

The strengths of this review are that it is systematic and it probes further than other reviews of SW-CRT studies via the use of thematic synthesis. Thematic synthesis depends on the quality of reporting of the included studies [48]. However, to our knowledge, our review provides the first steps in seeking an understanding of the range of SW-CRT-related problems and responses to these problems. Moreover, whilst our review is just a snapshot of the literature, guidance in qualitative evidence relies more on confidence in synthesized findings than comprehensiveness. Our themes are general and thus could also apply to non-health settings.

There are, however, some limitations in this review. The search period for our review commenced just after the publication of the CONSORT extension and so we may have included studies of trialists who were unable to adopt the CONSORT recommendations or who had yet to become aware of this document. Whilst our sample of SW-CRTs might not reflect an immediately contemporary sample, there are unlikely to have been significant shifts in this field and so our findings are likely still highly relevant. Given the number and breadth of studies included, we believe that it is likely that we reached the saturation point regarding the overarching themes. Methodological quality was not assessed, as identifying SW-CRT-related problems was not the objective of the included studies. We also did not evaluate the rigor or robustness of the reported solutions. The scale of SW-CRT problems occurring in SW-CRT studies was also unclear. Authors may not have necessarily reported all the problems that they encountered nor all the actions they took in response to the problems that arose.

### Recommendations for future research

The complexities of the SW-CRT design will continue to present challenges to trialists in the context of real-world pressures. We recommend that oversight committees should carefully monitor SW-CRTs for implementation issues, given the increased risk of intervention delays and data-collection problems compared with other trial designs, and that an item should be added to reporting guidelines to record challenging implementational issues encountered.

The fact that several trials were preceded by studies that aimed to test the SW-CRT design but the trial then encountered problems demonstrates the challenges with this study design. Although not reported in our review, external events include the COVID-19 pandemic, which may continue to have an impact on current trials. We are aware of at least one SW-CRT that was interrupted due to COVID-19 [49].

Our study shows that conducting a prior feasibility or pilot study to anticipate problems may not necessarily avoid recruitment problems. The authors of the previous post-CONSORT 2022 review [22] observed a higher proportion of recruitment problems in studies that had a prior feasibility or pilot study than in those that did not. They speculated that this counterintuitive result might be explained as pilot studies are carried out in more complicated studies that are expected to have more recruitment problems. We found a similar result in our review. Our review will inform further research to better understand the difficulties with SW-CRTs, how trialists respond to problems, and whether these responses have resource or integrity ramifications. Insight may be gained by conducting in-depth interviews of trialists to explore individual perspectives or involving trialists in a Delphi process to seek consensus about what responses work best. The findings from such studies will help to develop mitigating strategies. Whilst this would aim to produce general advice, future research could focus on tailoring advice to particular settings or other subgroups.

## Conclusion

As a first step in developing guidance, our systematic review provides insight into reported SW-CRT problems and responses to problems, probing further than do traditional systematic reviews of SW-CRTs. We present two conflicting overarching themes to explain the complexities underlying the reported problems. Our review was hampered by underreporting. We highlight the need for further investigation into SW-CRT problems and how to mitigate them or develop alternative designs.

## Ethics approval

None was required.

## Supplementary Material

dyaf217_Supplementary_Data

## Data Availability

Data are presented or reported in published articles, which are publicly available. In addition, data from several supplementary tables were merged into an Excel file to facilitate manual searching. This file is available from GitHub at https://github.com/Kathy-Taylor/SWD-SR.
