# Supplementary material for: Reported problems and responses during the conduct of stepped-wedge cluster randomized trials in healthcare settings: a qualitative systematic review
Source: Int J Epidemiol. 2026 Jan 2;55(1):dyaf217. doi: 10.1093/ije/dyaf217 (PMC12758014; doi:10.1093/ije/dyaf217)
Supplement: dyaf217_Supplementary_Data [file dyaf217_supplementary_data.docx]

**Reported problems and responses during the conduct of stepped-wedge cluster randomised trials in healthcare settings: a qualitative systematic review**

**Kathryn S Taylor^1^, Julie McLellan^1^, Caroline Kristunas^2^, Clare Bankhead^1^, Nicola Pidduck^1^, Nia W Roberts^3^, Rafael Perera^1^, Karla Hemming^4^**

**^1^** Nuffield Department of Primary Care Health Sciences, University of Oxford, UK; ^2^Institute of Cancer and Genomic Sciences, University of Birmingham, UK; **^3^** Bodleian Health Care Libraries, University of Oxford, UK; **^4^**Department of Public Health, Epidemiology and Biostatistics, University of Birmingham, UK.

SUPPLEMENTARY INFORMATION

Contents

[Methods: Search strategy and eligibility criteria 2](#_Toc207634363)

[Table S1. PRISMA 2020 for Abstracts Checklist 7](#_Toc207634364)

[Table S2. PRISMA 2020 Checklist 8](#_Toc207634365)

[Table S3. ENTREQ Checklist 11](#_Toc207634366)

[Table S4. References of studies included in the systematic review 14](#_Toc207634367)

[Table S5. Detailed characteristics of included stepped-wedge cluster randomised trials 19](#_Toc207634368)

[Table S6. Studies reporting stepped-wedge cluster randomised trial-related problems, with problem codes and themes 37](#_Toc207634369)

[Table S7. Studies reporting responses to stepped-wedge cluster randomised trial-related problems, with response code and themes 49](#_Toc207634370)

[References for supplementary file 55](#_Toc207634371)

# Methods: Search strategy and eligibility criteria

Search strategy

We modified a previously published electronic search strategy(1) by not imposing a language restriction and expanding the title, abstract and author keywords for "stepped wedge". Where available, methodological search filters were applied to restrict to randomised controlled trials(2, 3). To ensure a contemporary sample of trials, we considered a search period of over two years from the publication date of the CONSORT extension for SW-CRTs(4), 9 November 2018, until 23 February 2021. We aimed to increase the likelihood of including studies that report to a higher standard meeting these new CONSORT guidelines.

Searches were undertaken by an information specialist and conducted in MEDLINE(OvidSP)[1946-], EMBASE(OvidSP)[1974-], CINAHL(EBSCOHost)[1982-], PsycINFO(OvidSP)[1806-], Science Citation Index(Web of Science Core Collection)[1900-] and Cochrane Central Register of Controlled Trials(Cochrane Library, Wiley)[Issue 2 of 12, February 2021]. ClinicalTrials.gov (Classic) https://clinicaltrials.gov/ and WHO ICTRP https://trialsearch.who.int/ were searched for trial registrations. A forward citation search was conducted on Google Scholar, Scopus and Web of Science for publications citing the CONSORT extension to SW-CRTs. The reference lists of relevant reviews or commentaries were reviewed. The grey literature was not searched.

MEDLINE

1 (step* adj1 wedge).ti,ab,kw.

2 (swcrct or sw-crct).ti,ab,kw.

3 ((staged or delayed) adj (introduction or intervention)).ti,ab,kw.

4 (("one direction*" or "1 direction*") adj3 (crossover or cross over)).ti,ab,kw.

5 ((incremental or phased or stagger* or stepwise or step wise or delayed) adj1 (recruitment or introduction or implementation)).ti,ab,kw.

6 ((wait list? or waiting list?) adj1 (control? or crossover or cross over or design?)).ti,ab,kw.

7 1 or 2 or 3 or 4 or 5 or 6

8 randomized controlled trial.pt.

9 controlled clinical trial.pt.

10 randomized.ab.

11 placebo.ab.

12 drug therapy.fs.

13 randomly.ab.

14 trial.ab.

15 groups.ab.

16 8 or 9 or 10 or 11 or 12 or 13 or 14 or 15

17 exp animals/ not human/

18 16 not 17

19 7 and 18

20 limit 19 to yr="2018 -Current"

EMBASE

| 1 | (step* adj1 wedge).ti,ab,kw. |
| --- | --- |
| 2 | (swcrct or sw-crct).ti,ab,kw. |
| 3 | ((staged or delayed) adj (introduction or intervention)).ti,ab,kw. |
| 4 | (("one direction*" or "1 direction*") adj3 (crossover or cross over)).ti,ab,kw. |
| 5 | ((incremental or phased or stagger* or stepwise or step wise or delayed) adj1 (recruitment or introduction or implementation)).ti,ab,kw. |
| 6 | ((wait list? or waiting list?) adj1 (control? or crossover or cross over or design?)).ti,ab,kw. |
| 7 | 1 or 2 or 3 or 4 or 5 or 6 |
| 8 | randomized controlled trial/ |
| 9 | controlled clinical trial/ |
| 10 | random*.ti,ab. |
| 11 | randomization/ |
| 12 | intermethod comparison/ |
| 13 | placebo.ti,ab. |
| 14 | (compare or compared or comparison).ti. |
| 15 | ((evaluated or evaluate or evaluating or assessed or assess) and (compare or compared or comparing or comparison)).ab. |
| 16 | (open adj label).ti,ab. |
| 17 | ((double or single or doubly or singly) adj (blind or blinded or blindly)).ti,ab. |
| 18 | double blind procedure/ |
| 19 | parallel group*1.ti,ab. |
| 20 | (crossover or cross over).ti,ab. |
| 21 | ((assign$ or match or matched or allocation) adj5 (alternate or group$1 or intervention$1 or patient$1 or subject$1 or participant$1)).ti,ab. |
| 22 | (assigned or allocated).ti,ab. |
| 23 | (controlled adj7 (study or design or trial)).ti,ab. |
| 24 | (volunteer or volunteers).ti,ab. |
| 25 | human experiment/ |
| 26 | trial.ti. |
| 27 | 8 or 9 or 10 or 11 or 12 or 13 or 14 or 15 or 16 or 17 or 18 or 19 or 20 or 21 or 22 or 23 or 24 or 25 or 26 |
| 28 | (random$ adj sampl$ adj7 ("cross section$" or questionnaire$1 or survey$ or database$1)).ti,ab. not (comparative study/ or controlled study/ or randomi?ed controlled.ti,ab. or randomly assigned.ti,ab.) |
| 29 | Cross-sectional study/ not (randomized controlled trial/ or controlled clinical study/ or controlled study/ or randomi?ed controlled.ti,ab. or control group$1.ti,ab.) |
| 30 | (((case adj control$) and random$) not randomi?ed controlled).ti,ab. |
| 31 | (Systematic review not (trial or study)).ti. |
| 32 | (nonrandom$ not random$).ti,ab. |
| 33 | "Random field$".ti,ab. |
| 34 | (random cluster adj3 sampl$).ti,ab. |
| 35 | (review.ab. and review.pt.) not trial.ti. |
| 36 | "we searched".ab. and (review.ti. or review.pt.) |
| 37 | "update review".ti,ab. |
| 38 | (databases adj4 searched).ab. |
| 39 | (rat or rats or mouse or mice or swine or porcine or murine or sheep or lambs or pigs or piglets or rabbit or rabbits or cat or cats or dog or dogs or cattle or bovine or monkey or monkeys or trout or marmoset$1).ti. and animal experiment/ |
| 40 | Animal experiment/ not (human experiment/ or human/) |
| 41 | 28 or 29 or 30 or 31 or 32 or 33 or 34 or 35 or 36 or 37 or 38 or 39 or 40 |
| 42 | 27 not 41 |
| 43 | 7 and 42 |
| 44 | limit 43 to yr="2018-Current" |

PsycINFO

| 1 | (step* adj1 wedge).mp. |
| --- | --- |
| 2 | (swcrct or sw-crct).mp. |
| 3 | ((staged or delayed) adj (introduction or intervention)).mp. |
| 4 | (("one direction*" or "1 direction*") adj3 (crossover or cross over)).mp. |
| 5 | ((incremental or phased or stagger* or stepwise or step wise or delayed) adj1 (recruitment or introduction or implementation)).mp. |
| 6 | ((wait list? or waiting list?) adj1 (control? or crossover or cross over or design?)).mp. |
| 7 | 1 or 2 or 3 or 4 or 5 or 6 |
| 8 | random*.ti,ab,hw,id. |
| 9 | trial*.ti,ab,hw,id. |
| 10 | controlled stud*.ti,ab,hw,id. |
| 11 | placebo*.ti,ab,hw,id. |
| 12 | ((singl* or doubl* or trebl* or tripl*) and (blind* or mask*)).ti,ab,hw,id. |
| 13 | (cross over or crossover or factorial* or latin square).ti,ab,hw,id. |
| 14 | (assign* or allocat* or volunteer*).ti,ab,hw,id. |
| 15 | treatment effectiveness evaluation/ |
| 16 | mental health program evaluation/ |
| 17 | exp experimental design/ |
| 18 | (clinical trial or treatment outcome).md. |
| 19 | 8 or 9 or 10 or 11 or 12 or 13 or 14 or 15 or 16 or 17 or 18 |
| 20 | 7 and 19 |
| 21 | limit 20 to yr="2018 -Current" |

CINAHL

| S8 | S1 OR S2 OR S3 OR S4 OR S5 OR S6 Limiters - Published Date: 20180101-20211231; Clinical Queries: Therapy - High Sensitivity |
| --- | --- |
| S7 | S1 OR S2 OR S3 OR S4 OR S5 OR S6 |
| S6 | TI (("wait list*" or "waiting list*") N1 (control* or crossover or "cross over" or design*)) OR AB (("wait list*" or "waiting list*") N1 (control* or crossover or "cross over" or design*)) |
| S5 | TI ((incremental or phased or stagger* or stepwise or "step wise" or delayed) N1 (recruitment or introduction or implementation)) OR AB ((incremental or phased or stagger* or stepwise or "step wise" or delayed) N1 (recruitment or introduction or implementation)) |
| S4 | TX (("one direction*" or "1 direction*") N1 (crossover or cross over)) |
| S3 | TI ((staged or delayed) N1 (introduction or intervention)) OR AB ((staged or delayed) N1 (introduction or intervention)) |
| S2 | TI swcrct or sw-crct OR AB swcrct or sw-crct |
| S1 | TI (step* N1 wedge) OR (step* N1 wedge) OR AB (step* N1 wedge) OR (step* N1 wedge) |

CENTRAL

"#1 - ((step* NEAR/1 wedge)):ti,ab,kw OR ((swcrct or sw-crct)):ti,ab,kw OR (((staged or delayed) NEXT (introduction or intervention))):ti,ab,kw OR (((incremental or phased or stagger* or stepwise or "step wise" or delayed) NEAR/1 (recruitment or introduction or implementation))):ti,ab,kw OR ((("wait list*" or "waiting list*") NEAR/1 (control* or crossover or cross over or design*))):ti,ab,kw" with Publication Year from 2018 to 2021, in Trials

SCI

| # 4 | #2  AND  #1 Timespan=2018-2021 |
| --- | --- |
| # 3 | #2  AND  #1 |
| # 2 | TS=(random*  or  blind*  or  allocat*  or  assign*  or  trial*  or  placebo*  or  crossover*  or  cross-over*) |
| # 1 | **TITLE:**  (step* N1 wedge)  *OR*  **TOPIC:**  (swcrct or sw-crct)  *OR*  **TOPIC:**  (((staged or delayed)  NEXT  (introduction or intervention) ))  *OR*  **TOPIC:**  (((incremental or phased or stagger* or stepwise or "step wise" or delayed)  NEAR/1  (recruitment or introduction or implementation) ))  *OR*  **TOPIC:**  ((("wait list*" or "waiting list*")  NEAR/1  (control* or crossover or "cross over" or design*) )) |

ClinicalTrials.gov

| "step wedge" OR "stepped wedge" OR swcrct OR sw-crct OR "staged introduction" OR "staged intervention" OR "delayed introduction" OR "delayed intervention" OR "one direction crossover" OR "one direction cross over" \| First posted from 11/01/2018 to 02/23/2021 |
| --- |
| "incremental recruitment" OR "phased recruitment" OR "stagger recruitment" OR "staggered recruitment" OR "stepwise recruitment" OR "step wise recruitment" OR "delayed recruitment" \| First posted from 11/01/2018 to 02/23/2021 |
| "incremental introduction" OR "phased introduction" OR "stagger introduction" OR "staggered introduction" OR "stepwise introduction" OR "step wise introduction" \| First posted from 11/01/2018 to 02/23/2021 |
| "incremental implementation" OR "phased implementation" OR "stagger implementation" OR "staggered implementation" OR "stepwise implementation" OR "step wise implementation" OR "delayed implementation" \| First posted from 11/01/2018 to 02/23/2021 |
| "wait list control" OR "wait list crossover" OR "wait list cross over" OR "wait list design" \| First posted from 11/01/2018 to 02/23/2021 |
| "waiting list control" OR "waiting list crossover" OR "waiting list cross over" OR "waiting list design" \| First posted from 11/01/2018 to 02/23/2021 |

Eligibility criteria

We included reports of completed original research studies of all variants of SW-CRTs, including feasibility and pilot studies, with at least two steps (a step is where a new cluster receives the intervention) and with the cluster as the unit of randomisation. We only included completed studies and excluded any reports of ongoing SW-CRT studies. Also excluded papers / publications about SW-CRTs that are not actual reports of a SW-CRT; studies analysed as stepped-wedge designs retrospectively; studies self-described as non-randomised, quasi-experimental, partially randomised; and bi-directional cross-over designs; and before and after studies.

# Table S1. PRISMA 2020 for Abstracts Checklist

| **Section and Topic** | **Item #** | **Checklist item** | **Reported (Yes/No)** |
| --- | --- | --- | --- |
| **TITLE** | | |  |
| Title | 1 | Identify the report as a systematic review. | Yes |
| **BACKGROUND** | | |  |
| Objectives | 2 | Provide an explicit statement of the main objective(s) or question(s) the review addresses. | Yes |
| **METHODS** | | |  |
| Eligibility criteria | 3 | Specify the inclusion and exclusion criteria for the review. | Yes |
| Information sources | 4 | Specify the information sources (e.g. databases, registers) used to identify studies and the date when each was last searched. | Yes |
| Risk of bias | 5 | Specify the methods used to assess risk of bias in the included studies. | N/A  Modified thematic synthesis |
| Synthesis of results | 6 | Specify the methods used to present and synthesise results. | Yes |
| **RESULTS** | | |  |
| Included studies | 7 | Give the total number of included studies and participants and summarise relevant characteristics of studies. | Yes |
| Synthesis of results | 8 | Present results for main outcomes, preferably indicating the number of included studies and participants for each. If meta-analysis was done, report the summary estimate and confidence/credible interval. If comparing groups, indicate the direction of the effect (i.e. which group is favoured). | Yes  Meta analysis was not appropriate |
| **DISCUSSION** | | |  |
| Limitations of evidence | 9 | Provide a brief summary of the limitations of the evidence included in the review (e.g. study risk of bias, inconsistency and imprecision). | Yes |
| Interpretation | 10 | Provide a general interpretation of the results and important implications. | Yes |
| **OTHER** | | |  |
| Funding | 11 | Specify the primary source of funding for the review. | Yes |
| Registration | 12 | Provide the register name and registration number. | Yes |

*From:*  Page MJ, McKenzie JE, Bossuyt PM, Boutron I, Hoffmann TC, Mulrow CD, et al. The PRISMA 2020 statement: an updated guideline for reporting systematic reviews. BMJ 2021;372:n71. doi: 10.1136/bmj.n71. This work is licensed under CC BY 4.0.

# Table S2. PRISMA 2020 Checklist

| **Section and Topic** | **Item #** | **Checklist item** | **Location where item is reported** |
| --- | --- | --- | --- |
| **TITLE** | | |  |
| Title | 1 | Identify the report as a systematic review. | Title, page 5, supplementary methods |
| **ABSTRACT** | | |  |
| Abstract | 2 | See the PRISMA 2020 for Abstracts checklist. | Table S1 |
| **INTRODUCTION** | | |  |
| Rationale | 3 | Describe the rationale for the review in the context of existing knowledge. | pages 4-5 |
| Objectives | 4 | Provide an explicit statement of the objective(s) or question(s) the review addresses. | pages 2 and 5 |
| **METHODS** | | |  |
| Eligibility criteria | 5 | Specify the inclusion and exclusion criteria for the review and how studies were grouped for the syntheses. | supplementary methods |
| Information sources | 6 | Specify all databases, registers, websites, organisations, reference lists and other sources searched or consulted to identify studies. Specify the date when each source was last searched or consulted. | page 2, supplementary methods |
| Search strategy | 7 | Present the full search strategies for all databases, registers and websites, including any filters and limits used. | supplementary methods |
| Selection process | 8 | Specify the methods used to decide whether a study met the inclusion criteria of the review, including how many reviewers screened each record and each report retrieved, whether they worked independently, and if applicable, details of automation tools used in the process. | page 5 |
| Data collection process | 9 | Specify the methods used to collect data from reports, including how many reviewers collected data from each report, whether they worked independently, any processes for obtaining or confirming data from study investigators, and if applicable, details of automation tools used in the process. | page 6 |
| Data items | 10a | List and define all outcomes for which data were sought. Specify whether all results that were compatible with each outcome domain in each study were sought (e.g. for all measures, time points, analyses), and if not, the methods used to decide which results to collect. | N/A no outcome data |
|  | 10b | List and define all other variables for which data were sought (e.g. participant and intervention characteristics, funding sources). Describe any assumptions made about any missing or unclear information. | page 6 |
| Study risk of bias assessment | 11 | Specify the methods used to assess risk of bias in the included studies, including details of the tool(s) used, how many reviewers assessed each study and whether they worked independently, and if applicable, details of automation tools used in the process. | N/A  modified thematic analysis |
| Effect measures | 12 | Specify for each outcome the effect measure(s) (e.g. risk ratio, mean difference) used in the synthesis or presentation of results. | N/A |
| Synthesis methods | 13a | Describe the processes used to decide which studies were eligible for each synthesis (e.g. tabulating the study intervention characteristics and comparing against the planned groups for each synthesis (item #5)). | page 7 |
|  | 13b | Describe any methods required to prepare the data for presentation or synthesis, such as handling of missing summary statistics, or data conversions. | N/A |
|  | 13c | Describe any methods used to tabulate or visually display results of individual studies and syntheses. | N/A |
|  | 13d | Describe any methods used to synthesize results and provide a rationale for the choice(s). If meta-analysis was performed, describe the model(s), method(s) to identify the presence and extent of statistical heterogeneity, and software package(s) used. | page 7 |
|  | 13e | Describe any methods used to explore possible causes of heterogeneity among study results (e.g. subgroup analysis, meta-regression). | N/A |
|  | 13f | Describe any sensitivity analyses conducted to assess robustness of the synthesized results. | N/A |
| Reporting bias assessment | 14 | Describe any methods used to assess risk of bias due to missing results in a synthesis (arising from reporting biases). | N/A |
| Certainty assessment | 15 | Describe any methods used to assess certainty (or confidence) in the body of evidence for an outcome. | N/A |
| **RESULTS** | | |  |
| Study selection | 16a | Describe the results of the search and selection process, from the number of records identified in the search to the number of studies included in the review, ideally using a flow diagram. | Figure 1 |
|  | 16b | Cite studies that might appear to meet the inclusion criteria, but which were excluded, and explain why they were excluded. | N/A |
| Study characteristics | 17 | Cite each included study and present its characteristics. | Tables S4 and S5 |
| Risk of bias in studies | 18 | Present assessments of risk of bias for each included study. | N/A |
| Results of individual studies | 19 | For all outcomes, present, for each study: (a) summary statistics for each group (where appropriate) and (b) an effect estimate and its precision (e.g. confidence/credible interval), ideally using structured tables or plots. | N/A |
| Results of syntheses | 20a | For each synthesis, briefly summarise the characteristics and risk of bias among contributing studies. | N/A |
|  | 20b | Present results of all statistical syntheses conducted. If meta-analysis was done, present for each the summary estimate and its precision (e.g. confidence/credible interval) and measures of statistical heterogeneity. If comparing groups, describe the direction of the effect. | N/A |
|  | 20c | Present results of all investigations of possible causes of heterogeneity among study results. | N/A |
|  | 20d | Present results of all sensitivity analyses conducted to assess the robustness of the synthesized results. | N/A |
| Reporting biases | 21 | Present assessments of risk of bias due to missing results (arising from reporting biases) for each synthesis assessed. | N/A |
| Certainty of evidence | 22 | Present assessments of certainty (or confidence) in the body of evidence for each outcome assessed. | N/A |
| **DISCUSSION** | | |  |
| Discussion | 23a | Provide a general interpretation of the results in the context of other evidence. | pages 12-13 |
|  | 23b | Discuss any limitations of the evidence included in the review. | page 13-14 |
|  | 23c | Discuss any limitations of the review processes used. | page 13-14 |
|  | 23d | Discuss implications of the results for practice, policy, and future research. | page 12-14 |
| **OTHER INFORMATION** | | |  |
| Registration and protocol | 24a | Provide registration information for the review, including register name and registration number, or state that the review was not registered. | page 5 |
|  | 224b | Indicate where the review protocol can be accessed, or state that a protocol was not prepared. | page 5 |
|  | 24c | Describe and explain any amendments to information provided at registration or in the protocol. | N/A |
| Support | 25 | Describe sources of financial or non-financial support for the review, and the role of the funders or sponsors in the review. | page 15-16 |
| Competing interests | 26 | Declare any competing interests of review authors. | N/A. None are declared |
| Availability of data, code and other materials | 27 | Report which of the following are publicly available and where they can be found: template data collection forms; data extracted from included studies; data used for all analyses; analytic code; any other materials used in the review. | page 15 |

*From:*  Page MJ, McKenzie JE, Bossuyt PM, Boutron I, Hoffmann TC, Mulrow CD, et al. The PRISMA 2020 statement: an updated guideline for reporting systematic reviews. BMJ 2021;372:n71. doi: 10.1136/bmj.n71

# Table S3. ENTREQ Checklist

| No | Item | Guide and description | Page |
| --- | --- | --- | --- |
| 1 | Aim | State the research question the synthesis addresses | 2 and 5 |
| 2 | Synthesis methodology | Identify the synthesis methodology or theoretical framework which underpins the synthesis, and describe the rationale for choice of methodology (e.g. meta-ethnography, thematic synthesis, critical interpretive synthesis, grounded theory synthesis, realist synthesis, meta-aggregation, meta-study, framework synthesis). | 7 |
| 3 | Approach to searching | Indicate whether the search was pre-planned (comprehensive search strategies to seek all available studies) or iterative (to seek all available concepts until they theoretical saturation is achieved). | Supplementary methods |
| 4 | Inclusion criteria | Specify the inclusion/exclusion criteria (e.g. in terms of population, language, year limits, type of publication, study type). | Supplementary methods |
| 5 | Data sources | Describe the information sources used (e.g. electronic databases (MEDLINE, EMBASE, CINAHL, psycINFO, Econlit), grey literature databases (digital thesis, policy reports), relevant organisational websites, experts, information specialists, generic web searches (Google Scholar) hand searching, reference lists) and when the searches conducted; provide the rationale for using the data sources. | Supplementary methods |
| 6 | Electronic Search strategy | Describe the literature search (e.g. provide electronic search strategies with population terms, clinical or health topic terms, experiential or social phenomena related terms, filters for qualitative research, and search limits). | Supplementary methods |
| 7 | Study screening methods | Describe the process of study screening and sifting (e.g. title, abstract and full text review, number of independent reviewers who screened studies). | 5 |
| 8 | Study characteristics | Present the characteristics of the included studies (e.g. year of publication, country, population, number of participants, data collection, methodology, analysis, research questions). | 9, Table 1 and Table S5 |
| 9 | Study selection results | Identify the number of studies screened and provide reasons for study exclusion (e,g, for comprehensive searching, provide numbers of studies screened and reasons for exclusion indicated in a figure/flowchart; for iterative searching describe reasons for study exclusion and inclusion based on modifications to the research question and/or contribution to theory development). | 8, Figure 1 |
| 10 | Rationale for appraisal | Describe the rationale and approach used to appraise the included studies or selected findings (e.g. assessment of conduct (validity and robustness), assessment of reporting (transparency), assessment of content and utility of the findings). | N/A  modified thematic synthesis |
| 11 | Appraisal items | State the tools, frameworks and criteria used to appraise the studies or selected findings (e.g. Existing tools: CASP, QARI, COREQ, Mays and Pope [25]; reviewer developed tools describe the domains assessed: research team, study design, data analysis and interpretations, reporting). | N/A |
| 12 | Appraisal process | Indicate whether the appraisal was conducted independently by more than one reviewer and if consensus was required. | N/A  modified thematic synthesis |
| 13 | Appraisal results | Present results of the quality assessment and indicate which articles, if any, were weighted/excluded based on the assessment and give the rationale. | N/A  modified thematic synthesis |
| 14 | Data extraction | Indicate which sections of the primary studies were analysed and how were the data extracted from the primary studies? (e.g. all text under the headings “results /conclusions” were extracted electronically and entered into a computer software). | 6-8 |
| 15 | Software | State the computer software used, if any. | N/A |
| 16 | Number of reviewers | Identify who was involved in coding and analysis. | 7 |
| 17 | Coding | Describe the process for coding of data (e.g. line by line coding to search for concepts). | 7 |
| 18 | Study comparison | Describe how were comparisons made within and across studies (e.g. subsequent studies were coded into pre-existing concepts, and new concepts were created when deemed necessary). | 7 |
| 19 | Derivation of themes | Explain whether the process of deriving the themes or constructs was inductive or deductive. | 7 |
| 20 | Quotations | Provide quotations from the primary studies to illustrate themes/constructs, and identify whether the quotations were participant quotations of the author’s interpretation. | Tables S6 and S7 |
| 21 | Synthesis output | Present rich, compelling and useful results that go beyond a summary of the primary studies (e.g. new interpretation, models of evidence, conceptual models, analytical framework, development of a new theory or construct). | 9-12, Tables S6 and S7, Figure 2. |

# Table S4. References of studies included in the systematic review

| **Study ID** | **Reference** |
| --- | --- |
| Agarwal 2020 | Agarwal P, Kithulegoda N, Bouck Z, et al. Feasibility of an Electronic Health Tool to Promote Physical Activity in Primary Care: Pilot Cluster Randomized Controlled Trial. J Med Internet Res. 2020;22(2):e15424. |
| Aguis 2020 | Agius PA, Cutts JC, Han Oo W, et al. Evaluation of the effectiveness of topical repellent distributed by village health volunteer networks against Plasmodium spp. infection in Myanmar: A stepped-wedge cluster randomised trial. PLoS Med. 2020;17(8):e1003177. |
| Anger 2019 | Anger HA, Dabash R, Durocher J, et al. The effectiveness and safety of introducing condom-catheter uterine balloon tamponade for postpartum haemorrhage at secondary level hospitals in Uganda, Egypt and Senegal: a stepped wedge, cluster-randomised trial. Bjog. 2019;126(13):1612-21. |
| Barrera 2019 | Barrera R, Harris A, Hemme RR, et al. Citywide Control of Aedes aegypti (Diptera: Culicidae) during the 2016 Zika Epidemic by Integrating Community Awareness, Education, Source Reduction, Larvicides, and Mass Mosquito Trapping. J Med Entomol. 2019;56(4):1033-46. |
| Bersani 2020 | Bersani K, Fuller TE, Garabedian P, et al. Use, Perceived Usability, and Barriers to Implementation of a Patient Safety Dashboard Integrated within a Vendor EHR. Appl Clin Inform. 2020;11(1):34-45. |
| BEYOND-II | van Duinen-van den IJCL, Bakker C, Smalbrugge M, et al. Effects on staff outcomes from an intervention for management of neuropsychiatric symptoms in residents of young-onset dementia care units: A cluster randomised controlled trial. Int J Nurs Stud. 2019;96:35-43. |
| CATH TAG | Mitchell BG, Northcote M, Cheng AC, Fasugba O, Russo PL, Rosebrock H. Reducing urinary catheter use using an electronic reminder system in hospitalized patients: A randomized stepped-wedge trial. Infect Control Hosp Epidemiol. 2019;40(4):427-31. |
| CHIME | Sanfilippo KRM, McConnell B, Cornelius V, et al. Community psychosocial music intervention (CHIME) to reduce antenatal common mental disorder symptoms in The Gambia: a feasibility trial. BMJ Open. 2020;10(11):e040287. |
| CPACS-3 | Wu Y, Li S, Patel A, et al. Effect of a Quality of Care Improvement Initiative in Patients With Acute Coronary Syndrome in Resource-Constrained Hospitals in China: A Randomized Clinical Trial. (2380-6591 (Electronic)). |
| CRADLE-3 | Vousden N, Lawley E, Nathan HL, et al. Effect of a novel vital sign device on maternal mortality and morbidity in low-resource settings: a pragmatic, stepped-wedge, cluster-randomised controlled trial. Lancet Glob Health. 2019;7(3):e347-e56. |
| CSNAT-I | Lund L, Ross L, Petersen MA, et al. Effect of the Carer Support Needs Assessment Tool intervention (CSNAT-I) in the Danish specialised palliative care setting: a stepped-wedge cluster randomised controlled trial. BMJ Support Palliat Care. 2020. |
| DART | Kronman MP, Gerber JS, Grundmeier RW, et al. Reducing Antibiotic Prescribing in Primary Care for Respiratory Illness. Pediatrics. 2020;146(3). |
| DECIDE-LVAD | McIlvennan CK, Matlock DD, Thompson JS, et al. Caregivers of Patients Considering a Destination Therapy Left Ventricular Assist Device and a Shared Decision-Making Intervention: The DECIDE-LVAD Trial. JACC Heart Fail. 2018;6(11):904-13 |
| DIZZINCT | Kerber KA, Damschroder L, McLaughlin T, et al. Implementation of Evidence-Based Practice for Benign Paroxysmal Positional Vertigo in the Emergency Department: A Stepped-Wedge Randomized Trial. Ann Emerg Med. 2020;75(4):459-70. |
| DOSE HPV | Perkins RB, Legler A, Jansen E, et al. Improving HPV Vaccination Rates: A Stepped-Wedge Randomized Trial. Pediatrics. 2020;146(1). |
| ELISABETH | Freund Y, Cachanado M, Delannoy Q, et al. Effect of an Emergency Department Care Bundle on 30-Day Hospital Discharge and Survival Among Elderly Patients With Acute Heart Failure: The ELISABETH Randomized Clinical Trial. Jama. 2020;324(19):1948-56. |
| EPOCH | Peden CJ, Stephens T, Martin G, et al. A national quality improvement programme to improve survival after emergency abdominal surgery: the EPOCH stepped-wedge cluster RCT. |
| EvANtiPain | Raphaelis S, Frommlet F, Mayer H, Koller A. Implementation of a nurse-led self-management support intervention for patients with cancer-related pain: a cluster randomized phase-IV study with a stepped wedge design (EvANtiPain). BMC Cancer. 2020;20(1):559. |
| FallDem | Halek M, Reuther S, Müller-Widmer R, Trutschel D, Holle D. Dealing with the behaviour of residents with dementia that challenges: A stepped-wedge cluster randomized trial of two types of dementia-specific case conferences in nursing homes (FallDem). Int J Nurs Stud. 2020;104:103435. |
| Fasugba 2019 | Fasugba O, Cheng AC, Gregory V, et al. Chlorhexidine for meatal cleaning in reducing catheter-associated urinary tract infections: a multicentre stepped-wedge randomised controlled trial. Lancet Infect Dis. 2019;19(6):611-9. |
| Geldsetzer 2020 | Geldsetzer P, Bärnighausen K, Hettema A, et al. A stepped-wedge randomized trial and qualitative survey of HIV pre-exposure prophylaxis uptake in the Eswatini population. Sci Transl Med. 2020;12(562). |
| Graham 2019 | Graham HR, Bakare AA, Ayede AI, et al. Oxygen systems to improve clinical care and outcomes for children and neonates: A stepped-wedge cluster-randomised trial in Nigeria. PLoS Med. 2019;16(11):e1002951. |
| Gunnarsson 2019 | Gunnarsson R, Cullen P, Heal C, Banks J. Effect of promoting current local research activities on large monitors on the population's interest in health-related research: a randomised controlled trial. BMJ Open. 2019;9(8):e028714. |
| Haines 2020 | Haines TP, Palmer AJ, Tierney P, Si L, Robinson AL. A new model of care and in-house general practitioners for residential aged care facilities: a stepped wedge, cluster randomised trial. Med J Aust. 2020;212(9):409-15. |
| Haskins 2020 | Haskins L, Chiliza J, Barker P, et al. Evaluation of the effectiveness of a quality improvement intervention to support integration of maternal, child and HIV care in primary health care facilities in South Africa. BMC Public Health. 2020;20(1):318. |
| Healthy Hearts NYC | Shelley DR, Gepts T, Siman N, et al. Cardiovascular Disease Guideline Adherence: An RCT Using Practice Facilitation. (1873-2607 (Electronic)). |
| Heart Health NOW  (prev. FAST PACE NC) | Cykert S, Keyserling TC, Pignone M, et al. A controlled trial of dissemination and implementation of a cardiovascular risk reduction strategy in small primary care practices. Health Serv Res. 2020;55(6):944-53. |
| High-STEACS | Shah ASV, Anand A, Strachan FE, et al. High-sensitivity troponin in the evaluation of patients with suspected acute coronary syndrome: a stepped-wedge, cluster-randomised controlled trial. Lancet. 2018;392(10151):919-28. |
| ICAN QUIT in pregnancy | Bar-Zeev Y, Bovill M, Bonevski B, et al. Improving smoking cessation care in pregnancy at Aboriginal Medical Services: ‘ICAN QUIT in Pregnancy’ step-wedge cluster randomised study. BMJ Open. 2019;9(6):e025293. |
| IMPROVe-GAP | Lloyd M, Karahalios A, Janus E, et al. Effectiveness of a Bundled Intervention Including Adjunctive Corticosteroids on Outcomes of Hospitalized Patients With Community-Acquired Pneumonia: A Stepped-Wedge Randomized Clinical Trial. JAMA Intern Med. 2019;179(8):1052-60. |
| IRIS | Henrichs J, Verfaille V, Jellema P, et al. Effectiveness of routine third trimester ultrasonography to reduce adverse perinatal outcomes in low risk pregnancy (the IRIS study): nationwide, pragmatic, multicentre, stepped wedge cluster randomised trial. Bmj. 2019;367:l5517. |
| Keogh 2020 | Keogh S, Shelverton C, Flynn J, et al. Implementation and evaluation of short peripheral intravenous catheter flushing guidelines: a stepped wedge cluster randomised trial. BMC Medicine. 2020;18(1):252. |
| Ketema 2020 | Ketema L, Dememew ZG, Assefa D, et al. Evaluating the integration of tuberculosis screening and contact investigation in tuberculosis clinics in Ethiopia: A mixed method study. PLoS One. 2020;15(11):e0241977. |
| Leis 2020 | Leis JA, Powis JE, McGeer A, et al. Introduction of Group Electronic Monitoring of Hand Hygiene on Inpatient Units: A Multicenter Cluster Randomized Quality Improvement Study. Clin Infect Dis. 2020;71(10):e680-e5. |
| LIRE | Jarvik JG, Meier EN, James KT, et al. The Effect of Including Benchmark Prevalence Data of Common Imaging Findings in Spine Image Reports on Health Care Utilization Among Adults Undergoing Spine Imaging: A Stepped-Wedge Randomized Clinical Trial. JAMA Network Open. 2020;3(9):e2015713-e. |
| LOCoMOTive | Romijn A, Ravelli A, de Bruijne MC, et al. Effect of a cluster randomised team training intervention on adverse perinatal and maternal outcomes: a stepped wedge study. Bjog. 2019;126(7):907-14. |
| Making it Happen | van den Broek N, Ameh C, Madaj B, et al. Effects of emergency obstetric care training on maternal and perinatal outcomes: a stepped wedge cluster randomised trial in South Africa. BMJ Global Health. 2019;4(6):e001670. |
| Manz 2020 | Manz CR, Parikh RB, Small DS, et al. Effect of Integrating Machine Learning Mortality Estimates With Behavioral Nudges to Clinicians on Serious Illness Conversations Among Patients With Cancer: A Stepped-Wedge Cluster Randomized Clinical Trial. JAMA Oncology. 2020;6(12):e204759-e. |
| MaxART | Chao A, Spiegelman D, Khan S, et al. Mortality under early access to antiretroviral therapy vs. Eswatini's national standard of care: the MaxART clustered randomized stepped-wedge trial. HIV Med. 2020;21(7):429-40. |
| Mazurek 2020 | Mazurek MO, Parker RA, Chan J, Kuhlthau K, Sohl K. Effectiveness of the Extension for Community Health Outcomes Model as Applied to Primary Care for Autism: A Partial Stepped-Wedge Randomized Clinical Trial. JAMA Pediatr. 2020;174(5):e196306. |
| McGuiness 2020 | McGuinness SL, O'Toole J, Forbes AB, et al. A Stepped Wedge Cluster-Randomized Trial Assessing the Impact of a Riverbank Filtration Intervention to Improve Access to Safe Water on Health in Rural India. Am J Trop Med Hyg. 2020;102(3):497-506. |
| MONARCH | Yapa HM, De Neve JW, Chetty T, et al. The impact of continuous quality improvement on coverage of antenatal HIV care tests in rural South Africa: Results of a stepped-wedge cluster-randomised controlled implementation trial. PLoS Med. 2020;17(10):e1003150. |
| Naser 2020 | Naser AM, Doza S, Rahman M, et al. Consequences of access to water from managed aquifer recharge systems for blood pressure and proteinuria in south-west coastal Bangladesh: a stepped-wedge cluster-randomized trial. Int J Epidemiol. 2021;50(3):916-28. |
| Nepal Cookstove | Katz J, Tielsch JM, Khatry SK, et al. Impact of Improved Biomass and Liquid Petroleum Gas Stoves on Birth Outcomes in Rural Nepal: Results of 2 Randomized Trials. Glob Health Sci Pract. 2020;8(3):372-82. |
| NePeriQIP | Kc A, Ewald U, Basnet O, et al. Effect of a scaled-up neonatal resuscitation quality improvement package on intrapartum-related mortality in Nepal: A stepped-wedge cluster randomized controlled trial. PLoS Med. 2019;16(9):e1002900. |
| NICCI | Var C, Oberhelman RA, Shu T, et al. A Linked Community and Health Facility Intervention to Improve Newborn Health in Cambodia: the NICCI Stepped-Wedge Cluster-Randomized Controlled Trial. Int J Environ Res Public Health. 2020;17(5). |
| OPAL | Kirkham J, Maxwell C, Velkers C, Leung R, Moffat K, Seitz D. Optimizing Prescribing of Antipsychotics in Long-Term Care (OPAL): A Stepped-Wedge Trial. J Am Med Dir Assoc. 2020;21(3):381-7.e3. |
| ORCAS | Meya DB, Kiragga AN, Nalintya E, et al. Reflexive Laboratory-Based Cryptococcal Antigen Screening and Preemptive Fluconazole Therapy for Cryptococcal Antigenemia in HIV-Infected Individuals With CD4 <100 Cells/µL: A Stepped-Wedge, Cluster-Randomized Trial. J Acquir Immune Defic Syndr. 2019;80(2):182-9. |
| PACT-HF | Van Spall HGC, Lee SF, Xie F, et al. Effect of Patient-Centered Transitional Care Services on Clinical Outcomes in Patients Hospitalized for Heart Failure: The PACT-HF Randomized Clinical Trial. JAMA. 2019;321(8):753-61. |
| PAINRelieveIt Hospice | Wilkie DJ, Yao Y, Ezenwa MO, et al. A Stepped-Wedge Randomized Controlled Trial: Effects of eHealth Interventions for Pain Control Among Adults With Cancer in Hospice. J Pain Symptom Manage. 2020;59(3):626-36. |
| Palen 2019 | Palen TE, Sharpe RE, Jr., Shetterly SM, Steiner JF. Randomized Clinical Trial of a Clinical Decision Support Tool for Improving the Appropriateness Scores for Ordering Imaging Studies in Primary and Specialty Care Ambulatory Clinics. AJR Am J Roentgenol. 2019;213(5):1015-20. |
| PARROT | Duhig KE, Myers J, Seed PT, et al. Placental growth factor testing to assess women with suspected pre-eclampsia: a multicentre, pragmatic, stepped-wedge cluster-randomised controlled trial. Lancet. 2019;393(10183):1807-18. |
| PERCEIVE | Wykes T, Csipke E, Rose D, et al. Patient involvement in improving the evidence base on mental health inpatient care: the PERCEIVE programme. 2018;6:7. |
| PHRASYL | Straßner C, Noest S, Preussler S, et al. The impact of patient-held health records on continuity of care among asylum seekers in reception centres: a cluster-randomised stepped wedge trial in Germany. BMJ Global Health. 2019;4(4):e001610. |
| POC-EID | Sacks E, Cohn J, Ochuka B, et al. Impact of Routine Point-of-Care Versus Laboratory Testing for Early Infant Diagnosis of HIV: Results From a Multicountry Stepped-Wedge Cluster-Randomized Controlled Trial. J Acquir Immune Defic Syndr. 2020;84 Suppl 1(1):S5-s11. |
| Povu Poa School | Wichaidit W, Steinacher R, Okal JA, et al. Effect of an equipment-behavior change intervention on handwashing behavior among primary school children in Kenya: the Povu Poa school pilot study. BMC Public Health. 2019;19(1):647. |
| Pradhan 2019 | Pradhan E, Canning D, Shah IH, et al. Integrating postpartum contraceptive counseling and IUD insertion services into maternity care in Nepal: results from stepped-wedge randomized controlled trial. Reprod Health. 2019;16(1):69. |
| Pro-Motion | Nydahl P, Günther U, Diers A, et al. PROtocol-based MObilizaTION on intensive care units: stepped-wedge, cluster-randomized pilot study (Pro-Motion). Nurs Crit Care. 2020;25(6):368-75. |
| REMAIN HOME | Freeman CR, Scott IA, Hemming K, et al. Reducing Medical Admissions and Presentations Into Hospital through Optimising Medicines (REMAIN HOME): a stepped wedge, cluster randomised controlled trial. Med J Aust. 2021;214(5):212-7. |
| Rikin 2020 | Rikin S, Zhang C, Lipsey D, et al. Impact of an Opt-In eConsult Program on Primary Care Demand for Specialty Visits: Stepped-Wedge Cluster Randomized Implementation Study. J Gen Intern Med. 2020;35(Suppl 2):832-8. |
| SAMBA | Østerås N, Moseng T, van Bodegom-Vos L, et al. Implementing a structured model for osteoarthritis care in primary healthcare: A stepped-wedge cluster-randomised trial. PLoS Med. 2019;16(10):e1002949. |
| Schnipper 2021 | Schnipper JL, Samal L, Nolido N, et al. The Effects of a Multifaceted Intervention to Improve Care Transitions Within an Accountable Care Organization: Results of a Stepped-Wedge Cluster-Randomized Trial. J Hosp Med. 2021;16(1):15-22. |
| Selby 2019 | Selby NM, Casula A, Lamming L, et al. An Organizational-Level Program of Intervention for AKI: A Pragmatic Stepped Wedge Cluster Randomized Trial. J Am Soc Nephrol. 2019;30(3):505-15. |
| Shah 2020 | Shah L, Rojas Peña M, Mori O, et al. A pragmatic stepped-wedge cluster randomized trial to evaluate the effectiveness and cost-effectiveness of active case finding for household contacts within a routine tuberculosis program, San Juan de Lurigancho, Lima, Peru. Int J Infect Dis. 2020;100:95-103. |
| Shekhawat 2020 | Shekhawat NS, Niziol LM, Sharma SS, et al. The Utility of Routine Fundus Photography Screening for Posterior Segment Disease: A Stepped-wedge, Cluster-randomized Trial in South India. Ophthalmology. 2021;128(7):1060-9. |
| Short FRAT | Jellett J, Williams C, Clayton D, Plummer V, Haines T. Falls risk score removal does not impact inpatient falls: A stepped-wedge, cluster-randomised trial. J Clin Nurs. 2020;29(23-24):4505-13 |
| Silverberg 2020 | Silverberg ND, Otamendi T, Panenka WJ, et al. De-implementing Prolonged Rest Advice for Concussion in Primary Care Settings: A Pilot Stepped Wedge Cluster Randomized Trial. J Head Trauma Rehabil. 2021;36(2):79-86. |
| SMARThealth India | Peiris D, Praveen D, Mogulluru K, et al. SMARThealth India: A stepped-wedge, cluster randomised controlled trial of a community health worker managed mobile health intervention for people assessed at high cardiovascular disease risk in rural India. PLoS One. 2019;14(3):e0213708. |
| SmartRub | Pires D, Gayet-Ageron A, Guitart C, et al. Effect of Wearing a Novel Electronic Wearable Device on Hand Hygiene Compliance Among Health Care Workers: A Stepped-Wedge Cluster Randomized Clinical Trial. JAMA Netw Open. 2021;4(2):e2035331. |
| SOCLE II | Brady MC, Stott DJ, Weir CJ, et al. A pragmatic, multi-centered, stepped wedge, cluster randomized controlled trial pilot of the clinical and cost effectiveness of a complex Stroke Oral healthCare intervention pLan Evaluation II (SOCLE II) compared with usual oral healthcare in stroke wards. Int J Stroke. 2020;15(3):318-23. |
| SO-HIP | Pol MC, Ter Riet G, van Hartingsveldt M, Kröse B, Buurman BM. Effectiveness of sensor monitoring in a rehabilitation programme for older patients after hip fracture: a three-arm stepped wedge randomised trial. Age Ageing. 2019;48(5):650-7. |
| SPEC | Kim H, Jung Y-i, Kim G-S, Choi H, Park Y-H. Effectiveness of a Technology-Enhanced Integrated Care Model for Frail Older People: A Stepped-Wedge Cluster Randomized Trial in Nursing Homes. The Gerontologist. 2021;61(3):460-9. |
| STRAP | van de Maat JS, Peeters D, Nieboer D, et al. Evaluation of a clinical decision rule to guide antibiotic prescription in children with suspected lower respiratory tract infection in The Netherlands: A stepped-wedge cluster randomised trial. PLoS Med. 2020;17(1):e1003034 |
| STRIVE | Ward J, Guy RJ, Rumbold AR, et al. Strategies to improve control of sexually transmissible infections in remote Australian Aboriginal communities: a stepped-wedge, cluster-randomised trial. (2214-109X (Electronic)). |
| STUMBL | Battle C, Hutchings HA, Driscoll T, et al. A multicentre randomised feasibility STUdy evaluating the impact of a prognostic model for Management of BLunt chest wall trauma patients: STUMBL Trial. BMJ Open. 2019;9(7):e029187. |
| Suman 2019 | Suman A, Schaafsma FG, van Dongen JM, et al. Effectiveness and cost-utility of a multifaceted eHealth strategy to improve back pain beliefs of patients with non-specific low back pain: a cluster randomised trial. BMJ Open. 2019;9(12):e030879. |
| THISTLE | Lenguerrand E, Winter C, Siassakos D, et al. Effect of hands-on interprofessional simulation training for local emergencies in Scotland: the THISTLE stepped-wedge design randomised controlled trial. BMJ Qual Saf. 2020;29(2):122-34. |
| Training for life | van Tetering AAC, Segers MHM, Ntuyo P, et al. Evaluating the Instructional Design and Effect on Knowledge, Teamwork, and Skills of Technology-Enhanced Simulation-Based Training in Obstetrics in Uganda: Stepped-Wedge Cluster Randomized Trial. JMIR Med Educ. 2021;7(1):e17277. |
| Trent 2019 | Trent SA, Havranek EP, Ginde AA, Haukoos JS. Effect of Audit and Feedback on Physician Adherence to Clinical Practice Guidelines for Pneumonia and Sepsis. Am J Med Qual. 2019;34(3):217-25. |
| Williams 2019 | Williams A, Rushton A, Lewis JJ, Phillips C. Evaluation of the clinical effectiveness of a work-based mentoring programme to develop clinical reasoning on patient outcome: A stepped wedge cluster randomised controlled trial. PLoS One. 2019;14(7):e0220110. |
| WMM | Palermo TM, de la Vega R, Murray C, Law E, Zhou C. A digital health psychological intervention (WebMAP Mobile) for children and adolescents with chronic pain: results of a hybrid effectiveness-implementation stepped-wedge cluster randomized trial. Pain. 2020;161(12):2763-74. |
| Wong 2020 | Wong MWH, Xu YZ, Bone J, Srigley JA. Impact of patient and visitor hand hygiene interventions at a pediatric hospital: A stepped wedge cluster randomized controlled trial. Am J Infect Control. 2020;48(5):511-6. |
| Worster 2020 | Worster DT, Franke MF, Bazua R, et al. Observational stepped-wedge analysis of a community health worker-led intervention for diabetes and hypertension in rural Mexico. BMJ Open. 2020;10(3):e034749. |
| XPRES | Agizew T, Chihota V, Nyirenda S, et al. Tuberculosis treatment outcomes among people living with HIV diagnosed using Xpert MTB/RIF versus sputum-smear microscopy in Botswana: a stepped-wedge cluster randomised trial. BMC Infect Dis. 2019;19(1):1058. |

# Table S5. Detailed characteristics of included stepped-wedge cluster randomised trials

| **Study ID** | **Country** | **Setting** | **Study area** | **Rationale for stepped wedge design** | **Design A** | **Design B** | **Design C** | **ASS/SST** | **No of sequences** | **No of**  **clusters** |
| --- | --- | --- | --- | --- | --- | --- | --- | --- | --- | --- |
| Agarwal 2020*§ | Canada | Periodic health review at family health centre | Physical activity (PA) counselling | Only states ' a SW-CRT design is suitable to test the effect of an intervention on PA,' | 4 | 1 | 1 | 437/440 | 4 | 34 |
| Aguis 2020* | Myanmar | Community | Malaria | 'intervention necessitated implementation at the village level, the statistical performance of the design in terms of power (both within- and between-cluster variance is used), its capacity to model temporal effects, and the advantage of being able to deliver the intervention to all villages' | 2 | 2 | 2 | NR | 14 | 116 |
| Anger 2019 | Uganda, Egypt, Senegal | Hospital | Postpartum haemorrhage (PPH) | ‘we chose a stepped wedge design so that all sites eventually received the intervention’ | 4 | 1 | 1 | 60111/  43200 | 2 | 18 |
| Barrera 2019* | Puerto Rico | Community | Zika Epidemic | NR | 2 | 2 | 2 | NR | NA | 8 |
| Bersani 2020† | US | Acute care hospital | Patient safety | NR | 6 | NR | 2 | NR | NA | 12 |
| BEYOND-II | Netherlands | YOD Special Care Unit (SCUs) from nursing homes | Young onset dementia | 'assures that all clusters receive the care program and can benefit from the possible positive effects of the intervention' 'increases the motivation of care organizations to participate in the study' 'appropriate when there are practical and logistic constraints to implementing the intervention simultaneously to all participants in the intervention condition, which is applicable to this study' 'study power by enabling analyses between and within groups' | 1 | 2 | 1 | NR | 3 | 13 |
| CATH TAG* | Australia | Hospital | Urinary catheter | 'enables each ward to act as its own control, which mitigates the potential for some confounders such as variations in ward size and case mix. Staggered commencement and duration of the intervention supports feasibility while maintaining the rigour of the study' 'allow research staff to work with individual wards as they change over, maximising consistency of the intervention and aiding implementation' | 4 | 1 | 1 | 1167/  2100 | 5 | 10 |
| CHIME*§ | Gambia | Antenatal clinics | Pregnancy | 'Advantages over a parallel arm cluster trial include the requirement of a smaller sample size due to the availability of a within group comparison and prevention of potential disappointment for health clinics who are not randomised into the intervention' | 2 | 1 | 1 | 84/120 | 2 | 4 |
| CPACS-3 *† | China | Hospitals | Acute Coronary Syndrome | 'because it was anticipated that the study would be beneficial and receipt of the intervention was the strong preference of all participating hospitals and the government officials' in charge of the project. | 4 | 1 | 1 | 29346/ 20800 | 4 | 101 |
| CRADLE-3*†ǂ | India, Zimbabwe, Zambia, Sierra Leone, Uganda, Haiti, Malawi, Ethiopia | Maternity care. Primary, secondary and tertiary facilities | Maternity care | " because phased implementation across ten clusters was more feasible than simultaneous implementation and because it would have been challenging to achieve sufficient cluster matching." | 4 | 1 | 1 | 536233/  720000 | NA | 10 |
| CSNAT-I* | Denmark | Inpatient care at hospices or hospital SPC departments, outpatient care from these units in the home or in outpatient clinics. | Caregivers of specialised palliative care cancer patients | The SW-CRT design was chosen to account for variation between SPC units, to prevent contamination, and to make participation in the study attractive for SPC units as all units ultimately will deliver the intervention | 3 | 3 | 1 | 298/308 | 3 | 9 |
| DART* | US | Pediatric practices | Acute respiratory tract infections (ARTIs) | 'to maximize statistical power and allow each practice to receive the intervention through staggered implementation' | 2 | 2 | 1 | 57/48 | 4 | 19 |
| DECIDE-LVAD*†ǂ | US | Hospital | Heart Failure | 'This approach was chosen because the intervention engages clinicians and other program staff in addition to patients' | 2 | 1 | 1 | 385/168 | 4 | 6 |
| DIZZINCT*† | US | Hospital ED | Benign Paroxysmal Positional Vertigo (BPPV) | ‘The stepped-wedge design has the advantages of being acceptable to stakeholders when the intervention implements evidence-based practice, initiation of the intervention at multiple time points (when a single time point is not logistically feasible), and including contemporaneous controls to evaluate and adjust for secular trends'  ''because randomization at the provider level was not logistically feasible. The design also enabled contemporaneous controls with a small number of centers'  'the delivery of the intervention to all sites also had the advantage of enabling us to explore variation in implementation fidelity across the mix of sites.'  'some hospitals will contribute many more pre-intervention observations and this will allow us to observe for underlying secular trends to ensure that changes observed in care processes are attributable to the interventions.' | 4 | 1 | 1 | 7635/  1800 | NA | 5 |
| DOSE HPV*† | US | Primary clincial sites (1 x safety net hospital, rest health centres) | human papillomavirus (HPV) vaccination | 'to separate secular trends from intervention effects as well as incentivize participation by clinical sites because all sites received the intervention'  "Differential pre- and postintervention periods in the stepped-wedge design serve to control for secular trends and allow comparisons within sites over time as well as between sites’ | 4 | 1 | 2 | NR | 1 group of 2 sites, all the rest single clusters | 5 |
| ELISABETH* | France | Emergency Departments (EDs) | Acute heart failure | 'prevents contamination that could arise from a cluster cross-over design, as centers will first be allocated to standard care before implementing the intervention. Furthermore, a stepped-wedge design would also prevent a potential “period effect” that could have resulted from a simple before/after design' | 4 | 1 | 1 | 502/500 | NA | 15 |
| EPOCH* | UK | Hospitals | Emergency abdominal surgery | 'improve statistical power by facilitating within-cluster comparison'  'The design allows us to control adoption bias and adjust for time-based changes in the background level of patient care in the statistical analysis. A key strength of the stepped wedge design is that we can offer the quality improvement project to every site which takes part' | 4 | 1 | 2 | 15856/  27540 | 15 | 97 |
| EvANtiPain*† | Austria | Hospitals | Cancer-related pain | 'it allowed a sequential intervention rollout with corresponding before and after measures in each cluster given that recruitment is evenly distributed over time' | 2 | 1 | 2 | 355/323 | NA | 17 |
| FallDem*† | Germany | Nursing Homes | Dementia | 'Logistical and motivational constraints'  'enabled us to provide our intervention in 12 nursing homes and to control for the confounding effect of calendar time'  'important for the nursing homes to participate in the intervention. The introduction of case conferences (CC) was the main motivation for participating in the study.' | 3 | 3 | 1 | 465/360 | 10 | 12 |
| Fasugba 2019* | Australia | hospital | urinary tract infection | the stepped-wedge design was chosen for its feasibility. It also enabled each hospital to act as its own control, removing the potential for confounders such as variation in hospital size and casemix and differences between public and private hospitals | 6 | 1 | 1 | 1642/  2640 | NA | 3 |
| Geldsetzer 2020 | Eswatini | Primary care | HIV | 'it is statistically more efficient when the outcome is observed at the cluster rather than the individual level (32) and because the design has the advantage that all participating clusters receive the intervention during the study' | 4 | 1 | 1 | NR | 2 | 6 |
| Graham 2019* | Nigeria | Hospitals | Acute lower respiratory infection | ‘as the most efficient and pragmatic trial design, enabling all hospitals to receive the full oxygen system intervention over a period that was commensurate with the logistics of implementation.’ | 6 | 1 | 1 | 17143/  16770  children  7716/  7670  neonates | 4 | 12 |
| Gunnarsson 2019 | Australia | Emergency department waiting rooms | Public interest in public-health | NR | 4 | 1 | 1 | 1501/  1500 | NA | 2 |
| Haines 2020* | Australia | Residential aged care facilities | General health outcomes | NR | 6 | 3 | 1 | 15/15  facilities | 7 | 15 |
| Haskins 2020 | South Africa | Primary health clinic | HIV and maternal health | ‘provides a robust methodology for evaluation when a phased approach to implementation is appropriate… allows every cluster to provide pre and post intervention data over an extended period of time. This is a strong research design for implementation research where it is not feasible or acceptable to have a control group that does not receive the intervention. allows the benefits of providing the intervention to include all clusters we were able to learn from each intervention period and modify the intervention based on the previous sub-district’s experience [adaptive design]’ | 3 | 1 | 1 | 1441/  1350 | 4 | 27 |
| Healthy Hearts NYC* | US | Primary care practices | Cardiovascular disease | ‘This design offers several advantages to traditional cluster parallel designs, including the opportunity for all practice sites to receive the intervention.’ | 1 | 1 | 1 | 257/250 practices | 4 | 291 |
| Heart Health NOW  (prev. FAST P ACE NC) | US | Small primary care practices | Atherosclerotic  cardiovascular risk (ASCVD) | ‘The stepped-wedge design provided a rigorous control group that accounted for secular trends while allaying ethical concerns around withholding an intervention with potential benefit for a large, at-risk, population.’  ‘Strengths of our study include the stepped-wedge design that provided a control group to assure that the intervention was effective apart from secular trends, the pragmatic nature of the trial, the inclusion of over 200 practices, and the ability to discern a measurable effect in a population of over 140 000 patients.’ | 4 | 1 | 1 | NR | 6 | 292 |
| High-STEACS | Scotland | Secondary and tertiary care hosptials | Acute coronary syndrome | No explicit rationale given for stepped wedge design. ‘This was a pragmatic trial, and therefore we had to accept some flexibility in the implementation phase to accommodate shared out of- hours laboratory services, shared electronic patient records, and site closures (appendix)"- this is not a rationale for the design as the appendix says "For pragmatic reasons (shared lab facilities out of hours), the Vale of Leven and Royal Alexandra Hospital, Paisley were grouped and randomised together.’ | 6 | 1 | 1 | NR | 2 | 10 |
| ICAN QUIT in pregnancy  *†§ | Australia | Aboriginal medical services | Smoking in pregnancy | A step-wedge design was chosen since it allows the intervention to be delivered sequentially and therefore reduces the cost and burden of simultaneous implementation, while also providing some control of confounding factors through randomisation. This design will ensure all sites receive the intervention which is important from an ethical viewpoint 'all sites receive the intervention in a timelier fashion for example compared to a standard cluster RCT or wait-list intervention' | 3 and 1 | 2 and 1 | 1 | 45/30 HPs  22/10 women | 3 | 6 |
| IMPROVe-GAP | Australia | General internal medicine units | Community-Acquired Pneumonia | Its stepped-wedge randomised controlled trial design provides a means to address some significant ethical, organisational and other methodological challenges to evaluating the effectiveness of health-service interventions in complex hospital populations.  A stepped-wedge, cluster-randomized, controlled design enabled both phased implementation and the use of established statistical approaches to compare control and intervention groups while minimizing the potential for bias and confounding   This design is therefore analogous to an “upscaling” that effectively mimics the way an intervention might be deployed in practice and is therefore particularly well-suited to implementation and health-service research | 4 | 1 | 1 | 816/640 | 4 | 8 |
| IRIS*† | Netherlands | Midwifery practices | Ultrasonography in pregnancy | ‘The stepped wedge design reduced confounding owing to differences between midwifery practices because each practice applied the control and intervention strategy for some of the time.’ | 2 | 1 | 1 | 13046/ 15000 | 3 | 60 |
| Keogh 2020  * | Australia | Hospitals | Peripheral intravenous catheter (PIVC) flushing | ‘The SWCRT design was chosen over a traditional randomised controlled design due to an inability to minimise contamination between groups if randomising at a patient level. Further, the SWCRT design was preferred over the classic parallel-cluster design due to optimised feasibility and management of staggered rather than en bloc roll out of intervention.’ | 4 | 1 | 2 | NR | NA | 9 |
| Ketema 2020† | Ethiopia | Health centres | TB screening | ‘For the quantitative part, a stepped-wedge design was applied as this is an optimal design to evaluate phased in interventions’ | 4 | 1 | 1 | NR | 3 | 30 |
| Leis 2020* | Canada | Hospitals | Hand hygiene | NR | 2 | NA | 1 | na | 3 | 26 |
| LIRE*† | US | Hospital | Lumbar spine imaging | ‘We chose a stepped-wedge randomization because of the appeal of all clusters receiving the intervention by the end of the trial, facilitating implementation and the ability to perform both within-cluster (ie, before and after) and between-cluster comparisons.’ | 4 | 1 | 1 | NR | 5 | 100 |
| LOCoMOTive | Netherlands | Hospital | Obstetrics care | in a dynamic context for research the intervention was rolled out as planned, according to the stepped wedge design. Overall, the study has met the need for a multicentre longitudinal approach to investigate the effectiveness of a complex social intervention.  'To uphold a high quality of the intervention, this design was considered to be appropriate as the intervention periods in different clusters did not coincide' | 2 | 1 | 1 | NR | NA | 5 |
| Making it Happen*† | South Africa | Hospital | Obstetrics care | The stepped wedge cluster randomised trial design was chosen as it is an appropriate method for assessing interventions which are introduced into ‘routine’ services and which cannot be made available to all target sites simultaneously; the design allows each study site to act as its own control, while enabling any underlying secular effect of time on the outcomes to be accounted for ' 'We, however, recommend a stepped wedge trial as a feasible ‘real life’ evaluation of method of greater robustness than the more commonly conducted before–after comparisons in large-scale maternal and neonatal health programmes conducted in low-income and middle-income settings.' | 4 | 1 | 1 | NR | 11 | 129 |
| Manz 2020† | US | Oncology clinics | Conversations about cancer | Study strengths included ‘a stepped-wedge design that controlled for cancer site–specific uptake of SICs’ | 6 | 1 | 1 | NR | 4 | 8 |
| MaxART* | Eswatini | Public health sector | HIV | Protocol Walsh 2017 reports ‘A stepped-wedge randomized design is a causally strong and robust approach to determine if providing antiretroviral treatment for all HIV-positive individuals is a feasible intervention in a resource-limited, public sector health system.’ | 6 | 1 | 1 | 2960/  2088 | 7 | 14 |
| Mazurek 2020*† | US/Canada | Primary care physicians and practitioners | Autism | ‘this design allowed us to distribute limited resources to assess the effect of the ECHO Autism program across a broad range of sites chosen to maximize our ability to determine effectiveness of the intervention while minimizing potential contamination across groups and addressing potential ethical concerns. First, it would be problematic to randomize at the level of individual participants due to potential contamination across groups. Second, the staggered roll-out allows for comparison of each cluster to contemporaneous control groups as well as to its own baseline. Lastly, there will be a large benefit from the intervention for the participants themselves and we expect a large benefit for the patient populations that they serve as well.' | 1 | 2 | 2 | 148/150  PCPs | 5 | 10 |
| McGuiness 2020*† | India | Community | Diarrhoea prevention | ‘pragmatic study design in which all villages within the study eventually the intervention, thereby improving equity and acceptability. Comparisons between intervention and control are made within each cluster maximising statistical power particularly when cluster sizes are large. Between-cluster variation and seasonal effects are accommodated in the statistical analysis model.’ ‘for reasons of statistical power, budget, and funding duration’ | 1 | 2 | 2 | 1891/  1720 | NA | 4 |
| MONARCH | South Africa | Primary care clinics | Antenatal HIV care | ‘ (i) it was considered unethical to withhold the intervention from some clinics as CQI has known efficacy in resource-rich settings;  (ii) the participation of all clinics both during the control and the intervention exposure state was thought to be better if it was known that during the course of the trial all clinics would receive the intervention;  (iii) the field implementers of CQI were a small team of three people, making simultaneous rollout in all clusters impracticable; (iv) it allows adjustment for secular trends in outcomes.’ | 3 | 1 | 1 | 2160/  1260 | NA | 6 |
| Naser 2020*† | Bangladesh | Community | Blood pressure | ‘we took advantage of this roll-out plan…and implemented a stepped-wedge cluster-randomised controlled trial…thereby justifying further scale-up’  'allows communities to gradually have access to MAR water' 'each MAR site will contribute data for both the intervention and the control time periods' | 1 | 2 | 1 | 1191/  4668 | 4 | 16 |
| Nepal Cookstove | Nepal | Communities in rural Nepal | Pregnancy | NR | 4 | 2 | 1 | 2379/  2350 | 12 | 51 |
| NePeriQIP* | Nepal | Hospitals | Intrapartum care | ‘this pragmatic design allowed all clusters to be exposed to the intervention by the end of the study. …allows for the practical implementation of the intervention that would have been very resource demanding if it had been carried out at the same time at all hospitals’ | 6 | 1 | 1 | 88524 /  60000 | 4 | 12 |
| NICCI | Cambodia | Community | Newborn mortality | ‘so that all communities and facilities would receive the intervention, allows for robust statistical evaluation in resource-poor settings’ | 6 | 1 | 1 | 2494/  1760 | NA | 16 |
| OPAL | Canada | Long-term care (LTC) facility | Prescribing of antipsychotics | NR | 3 | 3 | 1 | NR | 4 | 10 |
| ORCAS* | Uganda | HIV clinics | HIV | ‘Chosen to accommodate the on-going adoption of WHO guidelines and the likely roll-out of CrAg screening in Uganda when the trial was initiated and to enable staggered training of clinical staff, laboratory personnel and pharmacists’ | 4 | 1 | 1 | 3359/  2190 | 5 | 9 |
| PACT-HF*† | Canada | Tertiary or quaternary care hospitals | Heart failure | ‘less subject to bias that typical quality improvement research methods; so that all hospitals received the intervention; because study outcomes were not available for several months after the trial's end, there would have been insufficient justification to implement the intervention among control sites had a parallel cluster design been used; the complexity of the work was simplified by initiating the intervention in a single cluster at each step; undesirable to recruit a large number of clusters at same time 'complexity of services in the PACT-HF trial made it undesirable to recruit a large number of clusters, and the desired number of clusters would have provided insufficient statistical power for a parallel cluster RCT to answer the primary research question.' 'is novel in cardiovascular research and less subject to bias than typical quality improvement research methods' | 6 | 1 | 1 | 2494/  3200 | NA | 10 |
| PAINRelieveIt Hospice* | US | Home | Cancer pain | NR | 4 | 1 | 1 | NR | 4 | 7 |
| Palen 2019 | US | Primary and speciality care ambulatory clinics | Imaging | ‘for practical reasons because the activation of the tool to clusters of medical office buildings prevented contamination of the intervention by ensuring separation of parties using and those not using the CDS tool at a specific point in time’ ‘allowed us to evaluate a rigorous, phased implementation of the CDS in a real-world setting.’ | 6 | 1 | 1 | NR | 4 | 23 |
| PARROT* | UK | Maternity units | Pre-eclampsia | ‘we chose a stepped-wedge design because we were aiming to introduce a biochemical test and associated management algorithm necessitating change in practice across a service. Women and investigators were clear that this’ ‘was their preferred study design because individual randomisation was perceived to be unfeasible, inequitable, and prone to contamination.’ | 5 | 1 | 1 | 1019/  514 | NA | 11 |
| PERCEIVE* | UK | Mental health trusts | Mental health | ‘As all clusters eventually receive the intervention, this design is often used in situations when, for ethical reasons, the researchers do not wish to deny the intervention to any particular cluster. This was one of the key considerations for the choice of this design’ | 3 | 3 | 1 | 1108/  315 | 8 | 16 |
| PHRASYL  *†ǂ | Germany | Reception centres | Patient held-health records | 'it was desired that all clusters should receive the intervention as soon as possible.' 'the pilot phase had provided evidence of the preliminary effectiveness of the PHR' ''a sequential rollout seemed to be more feasible than a rollout en bloc because the implementation of the PHR was a complex process comprising the elaboration of individual implementation concepts and instructions for the health personnel in the reception centres and the surrounding practices and clinics' | 4 | 3 | 1 | NR | 6 | 9 |
| POC-EID* | Kenya/Zimbabwe | District/ subdistrict hospitals/ primary care health facilities | Infant Diagnosis of HIV | NR | 4 | 1 | 1 | NR | NA | 3 |
| Povu Poa School*§ | Kenya | School | Hand washing | 'we chose the stepped-wedge design for several reasons. First, as the intervention posed no harm and brought benefit to the participating schools by creating places to wash hands with soap, it would be unethical to withhold the intervention in some participating schools. Second, the sequential roll-out of the intervention enabled the enumerators to deliver the intervention to the 30 participating schools while minimizing logistical constraints.' | 1 | 2 | 1 | NR | 3 | 30 |
| Pradhan 2019* | Sri Lanka/Nepal | Maternity care | Maternity care | ‘This allows us to estimate the impact of the intervention while controlling for underlying trends in PPIUD use’ (note this is a misconception!)  'potential benefit of the intervention to all women' (protocol) | 4 | 1 | 1 | 114650/  32400 | 2 | 6 |
| Pro-Motion  *§ | Germany | Hospital in-patient | Intensive care | Google translate from the protocol - Here everyone gets the intervention, however at different times. All clusters thus serve both as intervention and control group, however at different durations of study. Stepped wedge designs are suitable to complex investigating interventions | 3 | 1 | 1 | 272/360 | NA | 5 |
| REMAIN HOME* | Australia | **General practice** (P: people recently discharged from hospital with either a) 5+ long-term medications, b) congestive heart failure, c) exacerbation of COPD) | Hospital (re) admission Medication management | NR | 4 | 1 | 1 | 306/  2240 | 7 | 14 |
| Rikin 2020* | USA | Primary care | Demand for Specialty (secondary care) appointments | ‘We chose to scale our intervention using a stepped-wedge cluster randomized design to (1) quantify demand for eConsults over time to inform training needs for each eConsult speciality; (2) provide on-site training for each primary care practice during standing meetings; (3) evaluate the effect of our program on outcomes’ | 4 | 1 | 1 | NR | 3 | 16 |
| SAMBA* | Norway | Primary care | Osteoarthritis care (OA) | ‘was chosen since it allowed all clusters to test the intervention, and the GP and PT training could be done in one cluster at the time over a longer period.’ | 4 | 1 | 1 | 393/388 | NA | 6 |
| Schnipper 2021*†ǂ | USA | Primary care practice | Care transmisions from hospital to home | NR | 4 | 1 | 2 | 1657/ 1800 | Unclear | 16 |
| Selby 2019* | UK | Hospitals | Acute kidney injury (AKI) | ‘SWCRTs are well suited to pragmatic aspects of the rollout of complex interventions, ethical issues are avoided if concerns about withholding an intervention in the control arm exist, and efficient trial design is possible’ | 3 | 1 | 1 | 24059/  10850 | NA | 5 |
| Shah 2020* | Peru | Non-hospital healthcare centres | Tuberculosis (TB) | 'useful in this study due to the required widescale roll-out of this public health intervention' 'staggered intervention initiation times increase the flexibility of the stepped-wedge design in practice' 'The phased initiation is crucial to undertaking implementation across all 34 health centres all of which require individual training and monitoring which would not have been feasible in a full roll out or even a parallel CRCT.' 'The stepped-wedge pragmatic RCT is the most suited so all partners could benefit from high quality evidence, yet pragmatic utility.' 'As it is phased in, the monitoring, supervision and training can be improved and also any major problems, political, technical or resources can be identified to improve the implementation.' 'health centre NTP staff appeared motivated and engaged in training and preparation for the intervention once it is known that all centres will have to implement the new program and undergo the same intensive training, monitoring and evaluation processes, instead of some targeted added work assigned only to half of the centres' | 4 | 1 | 1 | NR | 4 | 34 |
| Shekhawat 2020*†ǂ | India | community eye clinics (vision centres VC) | Posterior segment eye diseases | ‘stepped-wedge trials enable measurement of an intervention’s effectiveness in real-world settings’ | 4 | 1 | 1 | NR | NA | 4 |
| Short FRAT | Australia | Hospital wards | Falls | NR | 3 | 1 | 1 | NR | 9 | 20 |
| Silverberg 2020* | Canada | Community | Advice for concussion | NR | 4 | 2 | 3 | 27/40 | NA | 7 |
| SMARThealth India*†ǂ | India | Primary care | Cardiovascular disease (CVD) | ‘will ensure that every participating PHC and village receives the intervention (for at least 6 months and an average of 12 months), while still allowing an unbiased evaluation of the effectiveness of the intervention compared to usual care.’ | 3 | 2 | 2 | 8642/  2700 | 3 | 18 |
| SmartRub* | Switzerland | Geriatric hospital | Hand hygiene (HH) | ‘stakeholders considered that the device use was a learning opportunity and thus should be made available for all study participants. Furthermore, the study team was limited by the availability of devices  and the sequential introduction of those in the wards was appropriate in terms of device production’ | 2 | 2 | 1 | 81/60 | 4 | 12 |
| SOCLE II*§ | UK | Stroke rehabilitation site | Oral Health care (OHC) in stroke patients | ‘Cluster randomisation was essential to minimise the risk of contamination. Logistical, financial and practical limitations prevented simultaneous delivery of the intervention to all sites. Providing the intervention across sites offered ethical and recruitment benefits’ | 4 | 1 | 1 | 325/325 patients  83/112 staff | NA | 4 |
| SO-HIP* | Netherlands | Nursing home/home | Rehabilitation after hip fracture | ‘1. Intervention effect can be estimated using between and within cluster comparisons and the professionals are their own controls in the interventions 2. Each participating nursing home will have implemented both interventions at the end of the study This increased nursing homes’ willingness to participate 3.Staggered start of the interventions makes a better time allowance (training, technical support) 4. Each participant receives only one condition, we may assume that there are no carryover effects’ | 4 | 1 | 1 | 240/288 | 3 | 6 |
| SPEC* | South Korea | nursing homes | Elder Care | ‘practical benefits for the research team, as well, with our limited human and financial resources’ | 3 | 3 | 2 | 525/450 | 5 | 10 |
| STRAP* | Netherlands | Emergency Department | lower respiratory tract infection *(RTI)* | ‘in general, smaller sample sizes are needed than in conventional cluster randomised trials. Because "clusters act as their own controls, the intervention effect can be estimated from both between and within cluster comparisons. The sequential implementation of the intervention was deemed superior to a conventional before–after design, given the incorporation of time effects’ | 4 | 1 | 1 | 912/900 | NA | 8 |
| STRIVE* | Australia | Primary health-care centres | Sexually transmissible infections (STI) | ‘ensured that all participating health centres would participate in the continuous quality improvement programme during the trial’ ‘Given that this design enables the intervention to be delivered to all clusters and that the intervention is likely to have a potential beneficial effect it was affirmed as a preferred model with key stakeholders’ | 3 | 3 | 2 | 24/14 | 3 | 24 |
| STUMBL  †ǂ | England/  Wales | Emergency department | Blunt chest wall trauma | NR | 6 | 1 | 1 | NR | NA | 4 |
| Suman 2019 | Netherlands | Primary healthcare practice | Nonspspecific low back pain (LBP) | ‘Because every cluster serves as its own control, but also as control for other clusters, this design allows both between group analyses as well as within-group analyses. Using this design, all clusters will have received the intervention at the end of the study, and thus, participation in this study will be higher this design allows the adaptation and improvement of the intervention strategy based upon experiences gathered during the stepwise implementation process.’ | 3 | 1 | 1 | 779/500 | NA | 4 |
| THISTLE* | Scotland | Maternity Units (with >900 births per year) | Intrapartum care | ‘there was not sufficient equipoise amongst participating centres about the intervention. So far, observational studies have consistently shown that PROMPT training is consistently associated with positive outcomes - indicating at least absence of clinical equipoise compared to no treatment and suggesting, albeit risk of confounding and selection biases associated with observational study design a potential treatment’ effectiveness. It was therefore deemed unethical to use a traditional parallel cluster RCT design in which some Maternity Units and their patients would have been allocated to no training. Furthermore, there was no obvious choice for an alternate training strategy to deliver in a control group as other similar training programmes have either been associated with harm or have no evidence with regards to their effectiveness and safety. Several Units had also requested the training prior to this study and the PROMPT training was supported by a Scottish Government initiative. A SW-RCT was considered as the safest and most ethical option allowing us to roll out the intervention to all Units whilst still using randomisation, and with a number of practical and logistic advantages including modest costs and staffing requirements. | 3 | 1 | 1 | 86514/  72000 | 3 | 12 |
| Training for life* | Uganda | Hospital | Obstetrics | ‘it is possible to control for time effects, and the design is useful when it is preferable to implement the intervention in stages because of logistical, practical or financial constraints’ ‘all clusters are trained’ | 6 | 2 | 1 | NR | NA | 7 |
| Trent 2019  *§ | US | Trauma center | Pneumonia and Sepsis | ‘studies that aim to change provider behavior are subject to attention bias and contamination, which can lead to overestimation of the intervention effect in before–after designs. The study team chose to mitigate this by using a stepped wedge design rather than a more simple before–after design to minimize such biases. ‘ | 4 | 1 | 1 | 469/915 | NA | 6 |
| Williams 2019*†ǂ | UK | 0utpatient departments | Musculo-skeletal physio-therapy | ‘for logistical and pragmatic reasons as this research was interested in the effects of this intervention being rolled out using outcomes for which it has not previously been considered, and the logistical and practical infeasibility of delivering this intervention (which is time and specialist ‘heavy’) in a parallel or crossover design’ | 1 | 1 | 1 | 323/432 | 3 | 6 |
| WMM | US | Clinics (pain and gastroenterology) | Chronic pain (pediatric) | ‘several advantages over alternatives such as a parallel group RCT, including (1) enhanced external validity, and (2) the ability to simultaneously test intervention effectiveness and collect implementation data." ‘testing in real world setting each cluster acts as control and intervention’ | 3 | 3 | 1 | 143/128 | 4 | 8 |
| Wong 2020* | Canada | Hospital units (inpatient units and emergency department) | Hand hygiene | NR | 3 | 3 | 1 | NR | 3 | 8 |
| Worster 2020* | Mexico | Community | Diabetes and hypertension | ‘practical approach for implementation and evaluation of low-risk interventions expected to confer a large benefit in impoverished settings, limiting the ethical issue of non-treatment common to randomised trials.’ 'allowed for individual-level analysis as in a stepped-wedge trial, limiting confounding by stable individual-level characteristics.' 'allowed us to rule out confounding by stable individual-level characteristics and adjust for underlying time trends' | 1 | 2 | 1 | NR | NA | 7 |
| XPRES*†ǂ | Botswana | HIV clinics | Tuberculosis screening in HIV patients | 'considered ethically sub-optimal to implement a parallel group CRT, where certain district tuberculosis (TB) labs and their associated clinics were denied access to Xpert for an extended period of time’  ‘the phased rollout of Xpert provided logistical advantages, because it meant that a single site activation team, in charge of training and activation of the Xpert device, could sequentially initiate all study sites’  ’ the need for only a single site activation team reduced projected study cost’ ‘program managers and funders were interested in assessing accuracy of the Xpert diagnostic algorithm in a real-world environment rather than trying to assess accuracy in a tightly controlled research environment, with limited external validity’ ‘in a real-world setting, the sequential rollout of an intervention allows lessons learned during earlier steps to be applied during later steps’  ’a stepped-wedge design provides analysis options that allow for the control of trends over time’ | 4 | 1 | 1 | 6041/  9614 | 9 | 22 |

Design A - Measurement of primary outcome

(1) Repeated measurements for participants at fixed calendar times, possibly linked to the timing of steps

(2) Repeated measurements for participants at times linked to the start of exposure, e.g. start and end of exposure

(3) Cross-sectional measurement, at fixed calendar time(s), possibly linked to timing of steps. Includes scenarios with repeated sampling at a low proportion from big clusters, so only a few individuals are sampled more than once

(4) Single measurement for each participant at a certain time after the start of their exposure

(5) Time-to-event, where time begins at the start of exposure

(6) Number of events in an exposure period. Note: A "participant" could be a patient or another unit of study.

Design B - Recruitment

(1) Continuous recruitment: Few (or even no) individuals participate as the trial begins, but more become eligible and participate over time, and are then exposed for a short period.

(2) Closed cohort: All participants are identified at the onset of the trial and participate from start till end.

(3) Open cohort: Substantial number of individuals are identified and participate from the start, but some may leave during the trial and others may become eligible. Minority of participants may also change between trial clusters. Possibly uses repeated measures or cross-sectional sampling

Design C – Data collection

(1) Complete: Intention to collect data from each cluster at all points throughout the trial

(2) Incomplete: Selected collection of data e.g. not collected in any lag period, collected only in certain periods before or after particular steps

Note: Lag period is the time between when a step and when the intervention can affect outcomes in participants.

* Reported a SW-CRT problem; † Pilot/feasibility study preceded the trial; ǂ Aim of prior pilot/feasibility study was to test the stepped wedge design; § SW-CRT was a pilot/feasibility study; NA - Not applicable; NR - Not reported; ASS/SST - Analysis sample size/sample size target

# Table S6. Studies reporting stepped-wedge cluster randomised trial-related problems, with problem codes and themes

| **Study** | **Reported problem** | **Problem codes** | **Problem descriptive theme** |  |
| --- | --- | --- | --- | --- |
| Aguis 2020 | ‘Of the original sample of 116 villages, 4 villages (3.4%) were excluded due to security concerns' | Security concerns in one or more clusters led to the loss of clusters  One or more clusters dropped out | External factors  Cluster issues |  |
| Barrera 2019 | 'Access to treatment varied among clusters, so that the actual sequence in which treatments was completed varied from the initial plan. An important aspect affecting treatment schedule and slowing down operations was the need to revisit structures at least three times before giving up because of residents’ absenteeism. Structures for which we did not receive authorization (absent res  ident, refusal) were excluded from treatment'. | Inconsistent intervention implementation across clusters  Absenteeism and lack of consent prevented intervention | Intervention implementation |  |
|  | ‘We anticipated the need for 100–110 field technicians to treat all eight clusters within 8 wk, but hiring and training such number of personnel was not possible... We also learned that contract field personnel needed more training in the use of maps and electronic devices to capture data. The field personnel hired to carry out vector control had no experience working for a vector control program nor previous entomologic training.' | Staff shortages, staff constraints or skill mix changes led to inconsistent implementation in one or more clusters | Internal factors |  |
| CATH TAG | 'evident that the research team was not able to obtain patient-day data that aligned precisely with the date when a ward switched from the control to the intervention' | Accuracy of timing of data collection at cross-over points | Outcome measurement |  |
|  | Did not meet recruitment target | Lower participant recruitment in one or more clusters | Participant recruitment |  |
| CHIME | ‘The timeline of the stepped-wedge schedule could not be strictly adhered to…….during Ramadan’ | Religious event in the control period shortened the study  Delayed or brought forward intervention rollout in one or more clusters | External factors  Intervention implementation |  |
|  | Did not meet recruitment target | Lower participant recruitment in one or more clusters | Participant recruitment |  |
| CRADLE-3† | Did not meet recruitment target | Lower participant recruitment in one or more clusters | Participant recruitment |  |
|  | ‘there were external changes within the site (strike action affecting staffing levels in three sites and natural disaster in Haiti’ | A natural disaster disrupted data collection  Industrial action in one or more clusters led to staff constraints  Staff constraints resulting from industrial action disrupting the intervention rollout | External factors  Internal factors |  |
| CPACS-3 | 'because of the technical constraints in hospitals at this level, many patients are often transferred to larger medical centers for better medical services. That would limit the ability for the intervention to take effect (there is not enough time for the intervention) and also prevent us from understanding the effect‘ | Some participants leave the cluster before outcome data is measurable  Medical services limitation at one or more clusters | Outcome measurement  Internal factors |  |
|  | One hospital from wedge 2 and 1 hospital from wedge 4 dropped out in cycle 2 | One or more clusters dropped out | Cluster issues |  |
| CSNAT-I | ‘was not optimal due to differences in the sizes of SPC teams …’ | Cluster size variation | Cluster issues |  |
|  | ‘…and thus minor modifications to the study design were required to ensure sufficient statistical power in analyses.’  Some clusters still in the control phase crossed over to the intervention earlier to boost overall recruitment in the intervention phase  ‘was not optimal due to .. and the slower enrolment rate during the intervention period…’ | None or lower participant recruitment in the intervention phase for one or more clusters  Delayed or brought forward intervention rollout in one or more clusters  Study duration was extended due to under recruitment | Participant recruitment  Intervention implementation  Internal factors |  |
| DART | '2 study clinicians each practiced at multiple practices that were randomized to different wedges.' | Staff working in multiple clusters due to resource constraints  Staff discussion between clusters leading to contamination | Internal factors  Intervention implementation |  |
| DECIDE-LVAD | ‘phased implementation randomized the site with the lowest LVAD implant rate to spend the most time in the intervention and the site with the highest LVAD implant rate to spend the most time in the control.' | Imbalance in eligible cases across the clusters between control and intervention | Participant recruitment |  |
| DIZZINCT | 'a relatively small number of randomization units and potential for intervention contamination because some providers worked at multiple sites.' | Staff discussion between clusters leading to contamination | Intervention implementation |  |
|  | The pre-rollout period was shortened, making the crossover point earlier and the step length longer for the first cluster by one month. This extended the roll-out, but there was no change to the study duration. Reason was not stated. | Delayed or brought forward intervention rollout in one or more clusters | Intervention implementation |  |
| DOSE HPV | ‘national change from 3-dose to 2-schedule’ | National guideline changes/differences in one or more clusters affected implementation  Inconsistent intervention implementation across clusters | External factors  Intervention implementation |  |
|  | 'Two of our sites transitioned to a new electronic medical record system, limiting our ability to identify patients who might receive primary care at the intervention sites but who had not presented for care in >12 months’ | Electronic health record system change in one or more clusters temporarily halted recruitment | Internal factors |  |
|  | This led to a 1-month delay in preintervention data collection in site 4 and an 8-month delay in site 5 and delays in the cross over times | Delayed or brought forward intervention rollout in one or more clusters  Delayed start due to internal factors | Intervention implementation  Internal factors |  |
| ELISABETH | 'acute heart failure seasonality and fewer cases in the summer period' | Lower participant recruitment in one or more clusters  Seasonal variation in the prevalence of the disease/condition led to the study being extended | Participant recruitment  External factors |  |
| EPOCH | ‘Hospitals which merged with other hospitals during the trial period,’ | Two clusters merged | Cluster issues |  |
|  | Did not meet recruitment target | Lower participant recruitment in one or more clusters | Participant recruitment |  |
| EvANtiPain | 'Recruitment was unevenly distributed over wards and hospitals……..29% of the wards, all within one hospital, recruited 68% of all participants……a larger number of patients in the control period.. the uneven distribution of observations over clusters by time ..The analysis of a stepped wedge design often includes a time trend, but this was not possible in our case due to the uneven distribution of observations over clusters by  time and the lack of control patients in 9 wards‘ | Imbalance in participant recruitment between clusters  Lower participant recruitment, particularly in the intervention phase | Participant recruitment  Participant recruitment |  |
|  | ‘…and the lack of control patients in 9 wards.’ | One or more clusters were exposed to the intervention for all steps | Intervention implementation |  |
|  | Paused implementation (to avoid summer holiday period when expected lower recruitment) and then extended the study to compensate (data collection)  ‘During the summer, recruitment was paused for two periods….recruitment was slower than expected’ | Lower participant recruitment in one or more clusters  Study duration was extended due to the paused implementation in some clusters when lower recruitment expected  Delayed or brought forward intervention rollout in one or more clusters | Participant recruitment  Internal factors  Intervention implementation |  |
| Fasugba 2019 | Did not meet recruitment target | Lower participant recruitment in one or more clusters | Participant recruitment |  |
| FallDem | 'Two NHs declined at T3 and T5 in the IdA cohort due to significant changes in leadership and unexpected increases in daily workload due to factors not related to the study (restructuring, staff shortage).' | One or more clusters dropped out  Staff shortages/skill mix changes in one or more clusters contributed to one of more clusters dropping out | Cluster issues  Internal factors |  |
|  | ‘The results could be impacted by measurement bias. Due to the SWD, the frequent data collection by care staff could influence the recall of information, sharpen attention to behaviour that challenges in daily work and reinforce the detection of this behaviour.’ | Measurement bias due to data collection by care staff | Outcome measurement |  |
| Graham 2019 | ‘we used a 2-week 'wash-in' period; however, actual practice change took longer’ | Actual practice change was longer than the planned transition period | Intervention implementation |  |
|  | ‘a lower-than-anticipated power to detect change in mortality outcomes (low event rates, low participant numbers, high intracluster correlation)’ 'the small number of clusters and the unbalanced cluster size limited the power of our study.’ | Cluster size variation  Higher than expected intra-cluster correlation  Low participant numbers  Higher participant recruitment in one or more clusters | Cluster issues  Participant recruitment |  |
|  | ‘Nigeria experienced a major economic recession, which impacted individuals, households, health facilities, and the broader health system. Participating government hospitals had extended periods of unpaid salaries, resulting in industrial action, hospital closure, increases in user fees, and deterioration in morale’ | Industrial action in one or more clusters disrupted the intervention rollout  One or more clusters closed | External factors  Cluster issues |  |
|  | ‘we excluded participants admitted during a 2-week ‘wash-in’ period at the beginning of each crossover period to implement the intervention and avoid contamination’ | Some participants were admitted during the (wash-in) transition period and were switched to intervention earlier than the rest of the cluster | Intervention implementation |  |
| Haines 2020 | [Noted by this review that several sites were not able to employ a GP for more than half of the 9-week time periods once they entered the intervention phase]  ‘four of the 15 sites (including three in regional cities) were unable to recruit a GP to work at their facility at any time during the stepped wedge trial and the prospective follow-up  periods’ | Inconsistent intervention implementation across clusters  Partially implemented intervention in one or more clusters  Staff shortages, staff constraints or skill mix changes led to inconsistent implementation in one or more clusters | Intervention implementation  Internal factors |  |
|  | ‘the stepped wedge design imposes a considerable data collection burden with its requirement to capture data during each time period of the investigation.’ | Measurement burden related to purposive data collection methods | Outcome measurement |  |
| Healthy Hearts NYC | ‘design also posed challenges in that all practice sites had to be recruited simultaneously and many had to wait several months to start the intervention.’  Primary recruitment challenges were identified: (1) out-of-date information about the practice; (2) lack of engagement with PCIP recruiters; and (3) competing practice priorities. | Frustration of having to wait for the intervention affected staff turnover  Staff shortages, staff constraints or skill mix changes caused recruitment challenges in one or more clusters  Lack of engagement with participant recruiters | Internal factors  Internal factors  Participant recruitment |  |
|  | ‘outcome data was not available from the 34 practices that withdrew after randomisation’ | One or more clusters dropped out | Cluster issues |  |
| ICAN QUIT in pregnancyǂ | ‘The variability around when the post survey was completed is another limitation, as changes in knowledge, attitudes and practices may change over time.’ | Variability across clusters of timing of post-intervention survey | Outcome measurement |  |
| IRIS | 'We did not achieve our required sample size of 15 000 women. Owing to the stepped wedge design, it was not possible to extend the study period because all midwifery practices had adopted the intervention strategy at the end of the study.’ | Lower participant recruitment in one or more clusters | Participant recruitment |  |
|  | 'After the first randomisation in April 2015, one midwifery practice withdrew from the study because of time constraints.' | One or more clusters dropped out | Cluster issues |  |
|  | ‘As recruitment was slower than anticipated, the predefined recruitment period of one year was extended, and hence the second group of midwifery practices crossed over to the intervention strategy one month later than planned’ | Lower participant recruitment in one or more clusters  Delayed or brought forward intervention rollout in one or more clusters | Participant recruitment  Intervention implementation |  |
| Keogh 2020 | The final 2 clusters received the intervention for the same period as the other clusters but after a delay during Dec/early Jan | Delayed or brought forward intervention rollout in one or more clusters  Study duration was extended due to under-staffing over the Christmas period | Intervention implementation  Internal factors |  |
| Leis 2020 | ‘One hospital (St Michael’s Hospital; 8 units) only randomized units to the first 2 dates due to tighter timeline for implementation’ | Some clusters had a shorter duration for the implementation of the intervention | Intervention implementation |  |
| LIRE | 'Two small clinics randomized to groups 2 and 5 were dropped before the first data submission because of clinic closure and are not included in the clinic counts' | One or more clusters dropped out | Cluster issues |  |
| Making it Happen | ‘Steps 7 & 8 were delayed due to logistic issues’ | Logistical/operational/administrative problems delayed the intervention rollout in one or more clusters  Delayed or brought forward intervention rollout in one or more clusters | Internal factors  Intervention implementation |  |
|  | 'The duration of the study and timing of the steps was such that two of the districts did not provide data for both the pre-intervention and post intervention periods which likely reduced the power of the study.' | One of more clusters did not provide intervention data as time ran out | Outcome measurement |  |
| MaxART | ‘guidelines evolved during the trial from recommending ART initiation at CD4<350 to CD4<500 and finally EAAA.’ | National guideline changes/differences in one or more clusters affected implementation  Inconsistent intervention implementation across clusters | External factors  Intervention implementation |  |
| Mazurek 2020 | ‘because of IRB [Institutional Review Board] or recruitment issues 2 sites started 1 month after the scheduled start of their cohort’ | Lower participant recruitment in one or more clusters  Approval delays led to a delayed intervention rollout in one or more clusters | Participant recruitment  Internal factors | |
|  | ‘Canadian guidelines for screening for autism were not consistent with US guidelines.’ [one cluster] | Inconsistent intervention implementation across clusters  National guideline changes/differences in one or more clusters affected implementation | Intervention implementation  External factors |  |
| McGuiness 2020 | ‘we observed that the last village to receive the intervention had the highest attrition rate and the lowest rate of intervention uptake, possibly reflective of frustration in having to wait for the intervention implementation…….This highlights the need to sustain participant interest and engagement during control periods, an aspect not specifically  targeted in our study.’ | Reduced participant engagement due to frustration of having to wait for the intervention  Frustration of having to wait for the intervention reduced intervention uptake | Participant recruitment  Intervention implementation |  |
|  | ‘heavy monsoonal flooding in northern Karnataka in mid-2016 delayed the construction of water distribution systems, which widened the gap between the start of enrolment and the start of the SW-CRT (start of T1) from 3 to 9 months.’ | Severe weather event delayed the study start  Environmental problems in one or more clusters led to delays which extended the study duration | External factors |  |
| Naser 2020 | Did not meet recruitment target | Lower participant recruitment in one or more clusters | Participant recruitment |  |
|  | ‘Three communities did not receive MAR access per the randomization schedule due to technical issues, including the presence of sand in the water’ | One or more clusters did not implement the intervention | Intervention implementation |  |
|  | ‘Deviating from the protocol, three communities (one each in steps 3,4 and 5) did not gain access to MAR water at the randomly assigned times because of ongoing groundwater-quality problems such as the presence of sand in the MAR water), which would have made it impractical to provide the water at that time. These three communities were not dropped from the study and consumed water from their usual water sources during the entire trial.’ | Environmental problems in one or more clusters disrupted the intervention rollout  Delayed or brought forward intervention rollout in one or more clusters | External factors  Intervention implementation |  |
| NePeriQIP | ‘at one hospital there was a long and unforeseen strike and factors like this paired with logistical considerations made it difficult to start at the same and intended dates of each wedge.’ | Industrial action in one or more clusters disrupted the intervention rollout  Delayed or brought forward intervention rollout in one or more clusters | External factors  Intervention implementation |  |
|  | ‘at one hospital there was a long and unforeseen strike and factors like this paired with logistical considerations made it difficult to start at the same and intended dates of each wedge.’ | Logistical/operational/administrative problems delayed the intervention rollout in one or more clusters  Delayed or brought forward intervention rollout in one or more clusters | Internal factors  Intervention implementation |  |
|  | ‘successive clusters were exposed to the diffusion of treatment effect, as the intervention was widely disseminated in the national forum and the single hospital-based QI study had received widespread attention.’ | Diffusion of treatment effect due to dissemination of intervention | Intervention implementation |  |
| ORCAS  † | ‘This analysis initially compared survival in the observational phase with survival in the interventional phase among persons enrolled in the 9 clinics during the stepped wedge, cluster-randomized trial. At the time of the second interim analysis, the DSMB recommended that CrAg screening be rolled out expeditiously at 8 additional clinics, which were to be randomized in the second year of the trial, following adoption of the CrAg screening intervention in the Uganda national guidelines.’ | National guideline changes/differences in one or more clusters affected implementation  Delayed or brought forward intervention rollout in one or more clusters  Inconsistent intervention implementation across clusters | External factors  Intervention implementation |  |
| PACT-HF | Did not meet recruitment target | Lower participant recruitment in one or more clusters | Participant recruitment |  |
| PAINRelieveIt Hospice | ‘numerous environmental context changes during the study. They included one hospice merging with two others…(teams were not changed), a new administration at the other hospice with subsequent process changes, a new hospice on the area significantly reducing referrals to one hospice and new institutional alliances changing referral patterns to both hospices.’ | Lower participant recruitment due to competition from a new external facility  Competition from a new external facility reduced recruitment in one or more clusters  Two or more clusters merged | Participant recruitment  External factors  Cluster issues |  |
|  | ‘unanticipated imbalance in study group size…..one team provided more referrals than the other teams… resulting in more control group participants than originally planned….. | Imbalance in participant recruitment between clusters  Higher participant recruitment in one or more clusters | Participant recruitment |  |
|  | ‘recruitment in some teams slowed over time, leading to a lower recruitment pace during the intervention stages.’ | Imbalance in participant recruitment between clusters  None or lower participant recruitment in the intervention phase for one or more clusters |  |  |
| PARROT | ‘We found a difference in recruitment numbers between the revealed and concealed testing groups due to higher  recruitment numbers in later blocks (when more sites had transitioned to revealed testing)…. It is possible that the increase in numbers recruited in the intervention group could be a form of selection bias in itself, with clinicians recruiting different women due to the test becoming available, or that revealing the test result could affect the diagnosis of pre-eclampsia itself.’ | Higher participant recruitment in later steps  Increased awareness of intervention over time in one or more clusters | Participant recruitment  Intervention implementation |  |
|  | ‘our intracluster correlation was 0.035 which was much lower than we allowed for in the power calculation (thereby increasing the study power). However the actual proportion of women diagnosed with pre-eclampsia was found to be lower than anticipated.’ | Lower than expected intra-cluster correlation | Cluster issues |  |
| PERCEIVE | ‘The original proposal was designed for the initial eight wards, but our Data Monitoring and Ethics Committee  suggested that we increase the numbers of wards (clusters) in order to improve generalisability and allow for more accurate modelling of the between-ward variation.’  ‘As the later eight wards joined the trial part way through it was not possible to collect outcome data prior to their entry into the study’ | Wanting to add more clusters after randomisation | Cluster issues |  |
| PHRASYL | ‘The reception centre Mannheim 2 was closed by the state authorities after 8 weeks so that no more data could be collected.’ | One or more clusters closed | Cluster issues |  |
| POC-EID | ‘There were fewer POC tests in Zimbabwe as compared to laboratory-based tests. Owing to the stepped-wedge nature of the design, most POC tests were performed in the later period of the study. Seasonal variations in testing demand may have contributed to this imbalance in testing. However, the reasons for this decrease in testing demand in the POC arm are not clear’ | Imbalance in participant recruitment between clusters  None or lower participant recruitment in the intervention phase for one or more clusters | Participant recruitment |  |
| Povu Poa School | ‘at post-intervention, approximately half of all observed handwashing systems still did not have water and soapy water available for handwashing.’ | Inconsistent intervention implementation across clusters | Intervention implementation |  |
| Pradhan 2019  [Sri Lanka] | ‘Rollout of insertion services also varied considerably over the study period and within the four hospitals with no prior history of providing PPIUD. For example, insertion services in Chilaw hospital were not provided for almost one year after the start of the intervention in the hospital due to delays in implementation, hospital administration changes, and frequent changes to PPIUD-trained medical staff (particularly medical residents in the hospital ward), who would be reassigned to other hospitals every 6 | Staff shortages, staff constraints or skill mix changes delayed the intervention rollout in one or more clusters  Logistical/operational/administrative problems delayed the intervention rollout in one or more clusters  Delayed or brought forward intervention rollout in one or more clusters | Internal factors  Intervention implementation |  |
|  | ‘As a result, women who came to Chilaw hospital for delivery would be counselled on PPIUD during the antenatal period but would not receive insertion services at the hospital.’ | Inconsistent intervention implementation across clusters | Intervention implementation |  |
| Pradhan 2019  [Nepal] | ‘Actual timing of the start of the intervention by hospitals in each group varied slightly due to the need for trainers to move between hospitals to provide training.' | Staff shortages, staff constraints or skill mix changes delayed the intervention rollout in one or more clusters  Staff working in multiple clusters due resource constraints  Delayed or brought forward intervention rollout in one or more clusters | Internal factors  Intervention implementation |  |
| Pro-motion | Did not meet recruitment target | Lower participant recruitment in one or more clusters | Participant recruitment |  |
| REMAIN HOME | Did not meet recruitment target | Lower participant recruitment in one or more clusters | Participant recruitment |  |
| Rikin 2020 | ‘We were unable to examine how different components of our implementation strategy impacted outcomes. For example, because of delays and variable timing of payments, we could not analyse the impact of incentives on eConsult utilization.’ | Inconsistent intervention implementation across clusters | Intervention implementation |  |
| SAMBA | Noted by this review that two steps were separated by a 4-month period, compared to 2 months between all the other steps, even though the protocol stated 2 months. No reason was given. | Delayed or brought forward intervention rollout in one or more clusters | Intervention implementation |  |
|  | 'the size of the groups is unbalanced due to a higher recruitment rate during the intervention phases of the study.’ | Lower participant recruitment in the control phase  Imbalance in participant recruitment between clusters | Participant recruitment |  |
| Schnipper 2021 | Inpatient pharmacist counselling was ‘not deployed on all units due to staffing constraints; Difficulties identifying patients in need of discharge counselling in time to provide it (i.e., before discharge)’  Inpatient discharge advocacy ‘not enough availability to see all patients’  14 out of 16 practices had a delayed implementation of the intervention labelled “delayed start”. 2 of these 14, part of the practice started the intervention on one date and part of the practice later | Staff shortages, staff constraints or skill mix changes delayed the intervention rollout in one or more clusters  Delayed or brought forward intervention rollout in one or more clusters | Internal factors  Intervention implementation |  |
|  | Did not meet recruitment target | Lower participant recruitment in one or more clusters | Participant recruitment |  |
| Selby 2019 † | ‘In September 2016, there was a computer systems failure of the laboratory information management system (LIMS) that served three of the participating hospitals. This meant that the AKI detection algorithm was not available, and laboratory data collection was not possible during this period. For this reason, the trial was extended to allow for an extra period of data collection (December 2016 to February 2017) so that the planned number of data collection blocks was achieved’ | Inconsistent intervention implementation across clusters  Study duration was extended due to computing systems failure and temporary intervention loss in some clusters | Intervention implementation  Internal factors |  |
| Shah 2020 | 'During crossover, there was a risk of contamination from the time the NTP staff were trained to implement the intervention prior to the clinic crossover date to the new program' | Intervention training times not consistent with intervention implementation  Intervention training in control period leading to contamination | Intervention implementation |  |
| Shekhawat 2020 | 'A fifth VC was originally selected to participate, but ultimately did not take part in stepped-wedge randomization and instead provided the intervention at all steps.' | One or more clusters were exposed to the intervention for all steps | Intervention implementation |  |
| Silverberg 2020 | ‘The stepped wedge design was pragmatic but required a lengthy overall study duration, which may have contributed to attrition as well as waning post-encounter form completion.’ | Waning interest due to the data collection burden | Internal factors |  |
|  | Did not meet recruitment target | Lower participant recruitment in one or more clusters | Participant recruitment |  |
| SMARThealth India | There was a strong heatwave in Andhra Pradesh with temperatures as high as 48 degrees…. in the second step of the trial during which two thirds of the cohort were in the control period and one third in intervention. There is a well-established inverse relationship between ambient temperatures and BP levels....’ | Severe weather event affected the prevalence of outcomes in some clusters | External factors |  |
| SmartRub | ‘we faced problems related to the production and delivery of the devices that induced delays in the pre-established dates of rollout from baseline to transition in some groups.’  This extended the rollout and study duration | Delayed or brought forward intervention rollout in one or more clusters  Production and delivery delays extended study duration | Intervention implementation  External factors |  |
| SOCLE II | ‘less than half the staff (41.1%) in the fourth site participated' | Inconsistent intervention implementation across clusters | Intervention implementation |  |
| SO-HIP | Did not meet recruitment target | Lower participant recruitment in one or more clusters | Participant recruitment |  |
| SPEC | ‘the implementation period and the switch between the control and intervention terms may have been about 1 or 2 weeks shorter or longer across the homes if more urgent operational situations came up’ | Logistical/operational/administrative problems delayed the intervention rollout in one or more clusters  Logistical/operational/administrative problems brought forward the intervention rollout in one or more clusters  Delayed or brought forward intervention rollout in one or more clusters | Internal factors  Intervention implementation |  |
| STRAP | ‘Logistical problems in starting the trial in 2 hospitals resulted in a longer baseline period before rollout' | Logistical problems delayed the study start | Internal factors |  |
|  | ‘Original protocol: Duration: 24 months, centers will be randomized to implementation of the intervention within the period of 6-15 months after starting date of the study. Change: Duration: 33 months, randomization to implementation of the intervention within 20 – 28 months after starting date of the study.’  ‘This period was chosen to take the seasonality of respiratory tract infections into account.’ | Seasonal variation in the prevalence of the disease/condition led to the study being extended | External factors |  |
| STRIVE | 'one health-care centre withdrawing before starting the intervention because of restricted capacity to participate; no data from this centre were used.' | One or more clusters dropped out | Cluster issues |  |
| THISTLE | ‘One eligible maternity unit that had attended PROMPT T3 training before the start of the study but had not commenced ‘in-house’ local PROMPT courses, was allocated to period 1 for inclusion in the study.’ | One cluster exposed to the intervention before the study | Intervention implementation |  |
|  | ‘Furthermore, maternity units were often unable to commence the intervention as planned, either starting earlier or later than the randomisation schedule.’ ‘While most units were keen to participate, adherence to the randomisation schedule was variable. Some units were able to commence training immediately after their T3 session and before their allocated step, whereas others started several months after the allocated starting point.’ | Logistical/operational/administrative problems delayed the intervention rollout in one or more clusters  Delayed or brought forward intervention rollout in one or more clusters | Internal factors  Intervention implementation |  |
|  | ‘The implementation of the intervention was very heterogeneous between units, with significant variation in the content of local courses, number of courses implemented and also the number of staff trained.’  ‘the intervention implementation was challenging and the restrictions on logistics, time and funding meant that we were unable to provide ongoing implementation support to the participating units, other than by phone or email.’ | Inconsistent intervention implementation across clusters  Staff shortages/skill mix changes in one or more clusters  Logistical/operational/administrative problems in one or more clusters  Lack of funding and resources let to inconsistent implementation across clusters | Intervention implementation  Internal factors |  |
|  | ‘Two units refused to implement the intervention | Refusal to implement the intervention by one or more clusters  One or more clusters did not implement the intervention | Intervention implementation |  |
| Training for life | ‘as it was not feasible to create working schedules with fixed teams including midwives, interns, SHOs, obstetricians, anaesthesiologists, and paediatricians’ | Staff working in multiple clusters due to resource constraints | Internal factors |  |
| Trent 2019 | Given that this study was performed at a single institution, contamination could have occurred if physicians who had received the intervention discussed the intervention with physicians who had not previously received the intervention.’ | Staff discussion between clusters | Intervention implementation |  |
|  | ‘The study was cut short by more than 1 month because of implementation of a new electronic health record system at the study site, which temporarily halted the ability to obtain data to analyze performance measures.' | Electronic health record system change in one or more clusters temporarily halted data collection which shortened the study duration | Internal factors |  |
|  | Did not meet recruitment target | Lower participant recruitment in one or more clusters | Participant recruitment |  |
| Williams 2019 | Extended one step and shortened the following step by one month. No reason was given. No change to the study duration | Delayed or brought forward intervention rollout in one or more clusters | Intervention implementation |  |
|  | Did not meet recruitment target | Lower participant recruitment in one or more clusters | Participant recruitment |  |
| Worster 2020 | ‘A lack of data that linked CHWs [community health workers] to patients also precluded adjustment for clustering at the CHW level, which would be important if some CHWs were more or less effective.’ | Cluster definition unclear due to poor linkage between observations and cluster | Cluster issues |  |
|  | ‘Organisational delay in roll-out for cohort 2 shortened the baseline pre-randomisation phase and resulted in a 3-month delay in implementation in community 7.' | Logistical/operational/administrative problems delayed the intervention rollout in one or more clusters  Delayed or brought forward intervention rollout in one or more clusters  Logistical problems delayed the study start | Internal factors  Intervention implementation |  |
|  | 'study size was limited by the number of patients meeting eligibility criteria in the communities where the intervention was to be implemented' | Lower participant recruitment in one or more clusters | Participant recruitment |  |
| Wong 2020 | ‘some of the staff who attended FLO sessions could have brought those ideas to the control or standard intervention units. This contamination effect would potentially reduce the differences in outcomes between the groups | Staff discussion between clusters leading to contamination | Intervention implementation |  |
| XPRES | ‘unable to control pre- and post-diagnostic test-related factors in the TB diagnostic cascade, such as delayed sputum sample submission, delayed sample transportation, lack of regular maintenance of GeneXpert instruments, and whether laboratories used the most recent version of the Xpert cartridge (G3 vs. G4).' | Intervention device update let to inconsistent intervention implementation  Inconsistent intervention implementation across clusters | External factors  Intervention implementation |  |
|  | Did not meet recruitment target | Lower participant recruitment in one or more clusters | Participant recruitment |  |

* Reported a response to the SW-CRT problem; † Study was included in the systematic review but only later identified as reporting a SW-CRT problem

# Table S7. Studies reporting responses to stepped-wedge cluster randomised trial-related problems, with response code and themes

| **Study** | **Reported problem** | - **Problem descriptive theme (s)** | **Reported response to the problem** | **Response descriptive theme** |
| --- | --- | --- | --- | --- |
| **DURING THE STUDY** |  |  |  |  |
| CATH TAG | 'evident that the research team was not able to obtain patient-day data that aligned precisely with the date when a ward switched from the control to the intervention' | - Outcome measurement | Changed the primary outcome | Changing outcomes |
| Aguis 2020 | ‘Of the original sample of 116 villages, 4 villages (3.4%) were excluded due to security concerns' | - External factors - Cluster issues | Added substitute clusters that were selected randomly | Adding new clusters |
| ORCAS | ‘This analysis initially compared survival in the observational phase with survival in the interventional phase among persons enrolled in the 9 clinics during the stepped wedge, cluster-randomized trial. At the time of the second interim analysis, the DSMB recommended that CrAg screening be rolled out expeditiously at 8 additional clinics, which were to be randomized in the second year of the trial, following adoption of the CrAg screening intervention in the Uganda national guidelines.’ | - Intervention implementation - External factors | Added clusters to the end of the study in a further randomisation wave | Adding new clusters |
| PERCEIVE | ‘The original proposal was designed for the initial eight wards, but our Data Monitoring and Ethics Committeesuggested that we increase the numbers of wards (clusters) in order to improve generalisability and allow for more accurate modelling of the between-ward variation.’  ‘As the later eight wards joined the trial part way through it was not possible to collect outcome data prior to their entry into the study’ | - Cluster issues | Added clusters to the end of the study in a further randomisation wave | Adding new clusters |
| Leis 2020 | ‘One hospital (St Michael’s Hospital; 8 units) only randomized units to the first 2 dates due to tighter timeline for implementation’ | - Intervention implementation | Modified randomisation to allow some clusters to have earlier crossover points and finish the study earlier | Modifying the randomisation |
| DECIDE LVAD | ‘phased implementation randomized the site with the lowest LVAD implant rate to spend the most time in the intervention and the site with the highest LVAD implant rate to spend the most time in the control.' | - Participant recruitment | Modified randomisation to allow the cluster with lower event rate to spend the most time in the intervention | Modifying the randomisation |
| IRIS | ‘As recruitment was slower than anticipated, the predefined recruitment period of one year was extended, and hence the second group of midwifery practices crossed over to the intervention strategy one month later than planned’ | - Participant recruitment - Intervention implementation | Intervention rollout was delayed in some clusters to boost recruitment in the control phase | Modifying the randomisation |
| CSNAT-I | “…and thus minor modifications to the study design were required to ensure sufficient statistical power in analyses.”  Some clusters still in the control phase crossed over to the intervention earlier to boost overall recruitment in the intervention phase | Participant recruitment  Intervention implementation   - Internal factors | Some clusters crossed over early to the intervention to boost recruitment | Modifying the randomisation |
| EvANtiPain | Paused implementation (avoided summer holiday period when expected lower recruitment) and then extended the study to compensate (data collection)  ‘During the summer, recruitment was paused for two periods….recruitment was slower than expected’ | Participant recruitment  Intervention implementation | Paused implementation when expected lower recruitment and then extended the study to compensate | Modifying the randomisation |
| DART | '2 study clinicians each practiced at multiple practices that were randomized to different wedges.' | - Internal factors - Intervention implementation | Classified all data as intervention data after clinicians' first exposure to the intervention | Modifying the randomisation |
| THISTLE | ‘One eligible maternity unit that had attended PROMPT T3 training before the start of the study but had not commenced ‘in-house’ local PROMPT courses, was allocated to period 1 for inclusion in the study.’ | - Intervention implementation | Cluster was allocated to a sequence in a non random way | Modifying the randomisation |
| Shah 2020 | 'During crossover, there was a risk of contamination from the time the NTP staff were trained to implement the intervention prior to the clinic crossover date to the new program' | - Intervention implementation | Reduced the duration of the rollout period | Reducing contamination |
| Trent 2019 | ‘Given that this study was performed at a single institution, contamination could have occurred if physicians who had received the intervention discussed the intervention with physicians who had not previously received the intervention.’ | - Intervention implementation | Instructed staff not to discuss the intervention with those working at other clusters | Reducing contamination |
| **DURING THE ANALYSIS** |  |  |  |  |
| CRADLE-3 | ‘there were external changes within the site (strike action affecting staffing levels in three sites and natural disaster in Haiti’ | - External factors - Internal factors | Removed four periods of data when external factors disrupted the intervention implementation in a sensitivity analysis | Modifying the analysis |
|  |  |  | Ignored the strike action and natural disaster and included all data in the main analysis | ‘Intention-to-treat’ |
| DECIDE LVAD | ‘phased implementation randomized the site with the lowest LVAD implant rate to spend the most time in the intervention and the site with the highest LVAD implant rate to spend the most time in the control.' | - Participant recruitment | Added a cluster random effect in the primary analysis | Modifying the analysis |
|  |  |  | Excluded the cluster random effect in a sensitivity analysis | Modifying the analysis |
| EPOCH | ‘Hospitals which merged with other hospitals during the trial period,’ | - Cluster issues | Included clusters in the analysis up to the point that they merged | ‘Intention-to-treat’ |
| EvANtiPain | 'Recruitment was unevenly distributed over wards and hospitals……..29% of the wards, all within one hospital, recruited 68% of all participants……a larger number of patients in the control period.. the uneven distribution of observations over clusters by time ..The analysis of a stepped wedge design often includes a time trend, but this was not possible in our case due to the uneven distribution of observations over clusters by  time and the lack of control patients in 9 wards‘ | - Participant recruitment - Internal factors | Did not adjust for time trends | Modifying the analysis |
| Graham 2019 | ‘we used a 2-week 'wash-in' period; however, actual practice change took longer’ | - Intervention implementation | Excluded participants admitted during a 2-week ‘wash-in’ transition period at the beginning of each crossover period to avoid contamination | Modifying the analysis  Reducing contamination |
| Haines 2020 | [Noted by this review that several sites were not able to employ a GP for more than half of the 9-week time periods once they entered the intervention phase]  ‘four of the 15 sites (including three in regional cities) were unable to recruit a GP to work at their facility at any time during the stepped wedge trial and the prospective follow-up periods’ | - Intervention implementation - Internal factors | Used an instrumental variable analysis | Modifying the analysis |
| Leis 2020 | ‘One hospital (St Michael’s Hospital; 8 units) only randomized units to the first 2 dates due to tighter timeline for implementation’ | - Intervention implementation | Ignored some clusters having an earlier crossover point | ‘Intention-to-treat’ |
| Making it happen | 'The duration of the study and timing of the steps was such that two of the districts did not provide data for both the pre-intervention and post intervention periods which likely reduced the power of the study.' | - Intervention implementation | Included data available (ran out of time) | ‘Intention-to-treat’ |
| Mazurek 2020 | ‘because of IRB [Institutional Review Board] or recruitment issues 2 sites started 1 month after the scheduled start of their cohort’ | - Participant recruitment - Internal factors | Ignored the delay in the crossover points for two clusters in the main analysis | ‘Intention-to-treat’ |
|  | ‘Canadian guidelines for screening for autism were not consistent with US guidelines.’ [one cluster] | - Participant recruitment - External factors | Ignored the differences in screening guidelines between clusters | ‘Intention-to-treat’ |
|  |  |  | Excluded the cluster that had used a different screening guideline in sensitivity analysis | Modifying the analysis |
| Naser 2020 | ‘Three communities did not receive MAR access per the randomization schedule due to technical issues, including the presence of sand in the water’  ‘Deviating from the protocol, three communities (one each in steps 3,  4 and 5) did not gain access to MAR water at the randomly assigned times because of ongoing groundwater-quality problems such as the presence of sand in the MAR water), which would have made it impractical to provide the water at that time. These three communities were not dropped from the study and consumed water from their usual water sources during the entire trial.’ | - Intervention implementation | Clusters that did not receive the intervention were considered in the control phase as a sensitivity analysis | Modifying the analysis |
|  |  |  | The data for the clusters that did not receive the intervention were analysed as if they had | ‘Intention-to-treat’ |
| NePeriQIP | ‘at one hospital there was a long and unforeseen strike and factors like this paired with logistical considerations made it difficult to start at the same and intended dates of each wedge.’ | - External factors - Intervention implementation | Ignored the implementation delays | ‘Intention-to-treat’ |
|  |  |  | Accounted for the implementation delays in a sensitivity analysis | Modifying the analysis |
| PERCEIVE | ‘The original proposal was designed for the initial eight wards, but our Data Monitoring and Ethics Committee  suggested that we increase the numbers of wards (clusters) in order to improve generalisability and allow for more accurate modelling of the between-ward variation.’  ‘As the later eight wards joined the trial part way through it was not possible to collect outcome data prior to their entry into the study’ | - Cluster issues | For clusters joining the trial late, control data was obtained from an appropriate cross-sectional study | Modifying the analysis |
| Selby 2019 | ‘In September 2016, there was a computer systems failure of the laboratory information management system (LIMS) that served three of the participating hospitals. This meant that the AKI detection algorithm was not available, and laboratory data collection was not possible during this period. For this reason, the trial was extended to allow for an extra period of data collection (December 2016 to February 2017) so that the planned number of data collection blocks was achieved’ | - Intervention implementation - Internal factors | Excluded data from the period when the intervention was not available | Modifying the analysis |
| Shekhawat 2020 | 'A fifth VC was originally selected to participate, but ultimately did not take part in stepped-wedge randomization and instead provided the intervention at all steps.'  ‘The stepped wedge design was pragmatic but required a lengthy overall study duration, which may have contributed to attrition as well as waning post-encounter form completion.’ | - Intervention implementation | Excluded the cluster that had no control period in the main analysis | Modifying the analysis |
|  |  |  | Included the cluster that had no control period in a sensitivity analysis | Modifying the analysis |
| STRAP | ‘Logistical problems in starting the trial in 2 hospitals resulted in a longer baseline period before rollout' | - Internal factors | Truncated the extended pre-rollout period in a sensitivity analysis | Modifying the analysis |
|  |  |  | Included data from the extended pre-rollout period in the main analysis | ‘Intention-to-treat’ |
| THISTLE | ‘..maternity units were often unable to commence the intervention as planned, either starting earlier or later than the randomisation schedule.’  ‘While most units were keen to participate, adherence to the randomisation schedule was variable. Some units were able to commence training immediately after their T3 session and before their allocated step, whereas others started several months after the allocated  starting point.’ | - Intervention implementation | Conducted an 'as implemented' sensitivity analysis | Modifying the analysis |
|  |  |  | The data for the clusters that did not receive the intervention were analysed according to the randomisation schedule | ‘Intention-to-treat’ |

# References for supplementary file

1. Martin J, Taljaard M, Girling A, et al. Systematic review finds major deficiencies in sample size methodology and reporting for stepped-wedge cluster randomised trials. *BMJ Open* 2016;**6**:e010166. <https://doi.org/10.1136/bmjopen-2015-010166>
2. Glanville J, Foxlee R, Wisniewski S, et al. Translating the Cochrane EMBASE RCT filter from the Ovid interface to Embase.com: a case study. *Health Info Libr J* 2019;**36**:264-77. <https://doi.org/10.1111/hir.12269>
3. Lefebvre C, Glanville J, Briscoe S, et al. Technical Supplement to Chapter 4: Searching for and selecting studies [last updated September 2024]. In: Higgins JP, Thomas J, Chandler J, et al, (eds). *Cochrane Handbook for Systematic Reviews of Interventions* version 6.5. Cochrane, 2024. Available from <https://www.cochrane.org/authors/handbooks-and-manuals/handbook/current/chapter-04> (23 November 2025, date last accessed)
4. Hemming K, Taljaard M, McKenzie JE, et al. Reporting of stepped wedge cluster randomised trials: extension of the CONSORT 2010 statement with explanation and elaboration. *BMJ* 2018;**363**:k1614. <https://doi.org/10.1136/bmj.k1614>
